# cGMP Signalling Mediates Water Sensation (Hydrosensation) and Hydrotaxis in *Caenorhabditis elegans*

**DOI:** 10.1038/srep19779

**Published:** 2016-02-19

**Authors:** Wei Wang, Li-Wei Qin, Tai-Hong Wu, Chang-Li Ge, Ya-Qian Wu, Qiang Zhang, Yan-Xue Song, Yuan-Hua Chen, Ming-Hai Ge, Jing-Jing Wu, Hui Liu, Yao Xu, Chun-Ming Su, Lan-Lan Li, Jing Tang, Zhao-Yu Li, Zheng-Xing Wu

**Affiliations:** 1 Key Laboratory of Molecular Biophysics of Ministry of Education, Institute of Biophysics and Biochemistry, and Department of Biophysics and Molecular Physiology, College of Life Science and Technology, Huazhong University of Science and Technology, Wuhan, 430074, P.R. China

## Abstract

Animals have developed the ability to sense the water content in their habitats, including hygrosensation (sensing humidity in the air) and hydrosensation (sensing the water content in other microenvironments), and they display preferences for specific water contents that influence their mating, reproduction and geographic distribution. We developed and employed four quantitative behavioural test paradigms to investigate the molecular and cellular mechanisms underlying sensing the water content in an agar substrate (hydrosensation) and hydrotaxis in *Caenorhabditis elegans*. By combining a reverse genetic screen with genetic manipulation, optogenetic neuronal manipulation and *in vivo* Ca^2+^ imaging, we demonstrate that adult worms avoid the wetter areas of agar plates and hypo-osmotic water droplets. We found that the cGMP signalling pathway in ciliated sensory neurons is involved in hydrosensation and hydrotaxis in *Caenorhabditis elegans*.

Water is an important component of organisms and is essential for life. The environmental water content affects animals’ comfort, physiological homeostasis, behaviour, reproduction, survival and evolution. Mammals, birds, reptiles and insects have developed the ability to sense the water content in air (hygrosensation) and other microenvironments (hydrosensation) and display hygrotaxis or hydrotaxis^1^,^2^,^3^. Insects^4^, including *Drosophila*^1^,^2^,^5^; frogs^6^; lizards; toads^7^; rats^8^ and cats^9^ have sensory neurons that sense the external or internal water content that may, in turn, affect cellular osmolality, volume and stress^10^. Logically, both mechanosensation and hypo-osmosensation are involved in sensing water. The genes encoding the transient receptor potential (TRP) channels *water witch* and *nanchung*^2^ and the degenerin/epithelial sodium channels *pickpocket28*^1^,^11^ and *pickpocket1*^12^ in *Drosophila* and the putative mechanosensitive complex ASIC-1/MEC-10/MEC-6 and cGMP-gated channels in the thermosensitive AFD neurons in *C. elegans*^13^ are reported to function in hygrosensation and hygropreference. *C. elegans* is sensitive to desiccation. In soil, these worms live at an air-water interface and are exposed to both air and water^14^. Starvation-humidity levels paired worms can sense the humidity in the air (hygrosensation) *via* mechanosensation and thermosensation^13^. *C. elegans* reverses less frequently on drier agar plates^15^, suggesting that these worms may be sensitive to the water content in the substrate. However, whether the worms are able to detect the water content or its gradient in the substrate must be determined, and the underlying mechanisms remain an open question.

In this study, we demonstrate that adult *C. elegans* avoids both the wetter areas and hypo-osmotic water droplets in agar plates. We term this sensory modality hydrosensation to emphasise that the worms sense the water content in the substrate. We identify that the GPCR—G-protein—cGMP cyclase (DAF-11)—cGMP—cGMP-gated channel (TAX-2/TAX-4) signalling pathway in the ciliated sensory neuron pairs ASI, ASK and ASJ is involved in hydrosensation and hydrotaxis in *C. elegans*.

## Results

### *C. elegans* exhibits water avoidance (hydroavoidance)

**Figure 1:**
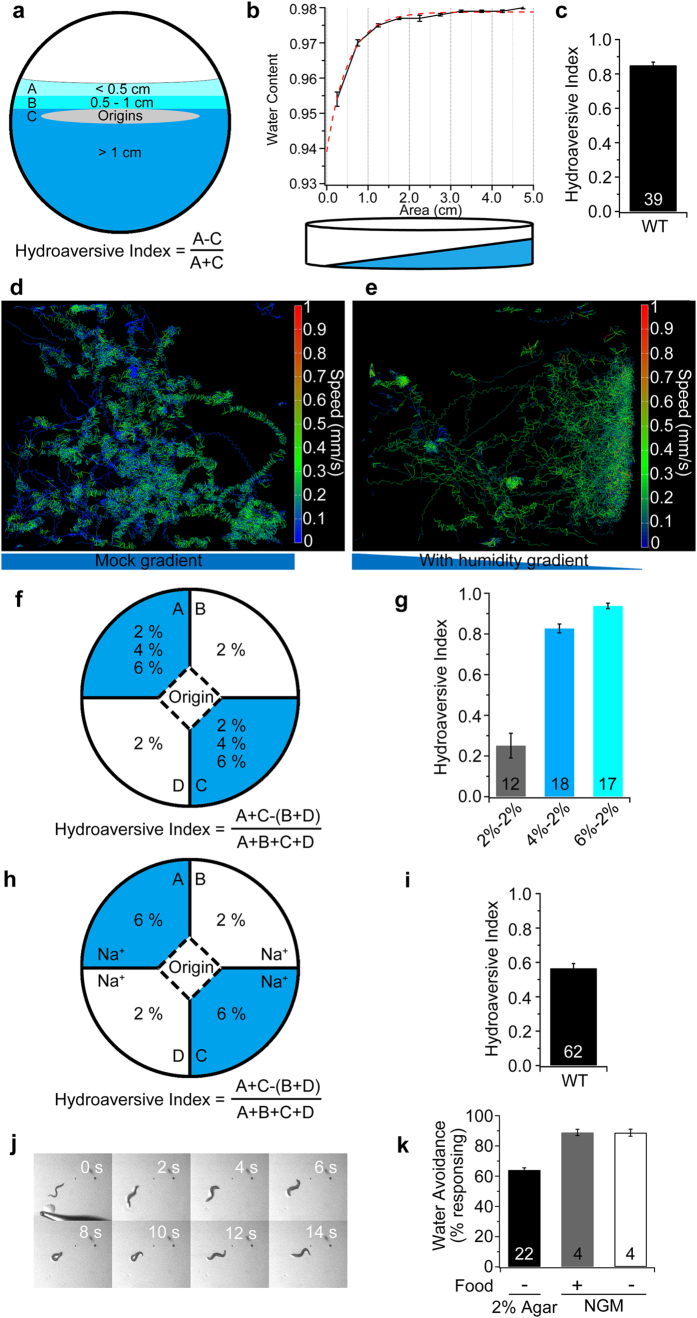
N2 worms exhibit hydropreference in multiple behavioural test paradigms. (**a**) Scheme of the wedge-shaped agar (WSA) test. Figure not drawn to scale. (**b**) Water content of the WSA plates. The dashed red line indicates the exponential fitted curve of the water content. (**c**) Hydroaversive index (H.A.) of the N2 worms in the WSA assay. (**d,e**) Trajectories of the N2 animals on regular agar (**d**) and WSA plates (**e**). (**f**) Scheme of the quadrant test. Figure not drawn to scale. (**g**) N2 worm hydrotaxis in the quadrant agar test. (**h**) Scheme of the four-quadrant agarose assay with equal concentrations of Na^+^. Figure not drawn to scale. (**i**) N2 worm hydrotaxis in the quadrant agarose test. (**j**) The snapshot images show a worm’s aversive response to a water drop. (**k**) H.A. index of the N2 worms in the drop test in different conditions. WT, wild-type N2. The number of independent tests is indicated for each test on the bar.

Under standard laboratory conditions, worms are usually cultured on 2% nematode growth medium (NGM) agar plates. We measured the average water content of NGM as 95.8%, and it varied from 97.4% to 92.7% with culture time (data not shown). To investigate whether *C. elegans* can sense the water content of agar substrates, we developed and used four behavioural paradigms to examine hydrosensation and hydrotaxis in the worm based on changing the water content of the medium. The main assay that we developed was the wedge-shaped agar (WSA) test (Fig. 1a and Methods, behavioural assay 1), in which the agar plate contains a continuous gradient of water content from the thick to the thin area that declines dramatically in the thin area (Fig. 1b). The wild-type N2 worms showed a preference for the drier area, *i.e.*, hydroavoidance (Fig. 1c). This hydroavoidance (negative hydrotaxis) was visualised by monitoring the worms’ locomotion on 2.0 cm × 2.5 cm pieces of uniform and wedge-shaped agar plates (Fig. 1d and e, Supplementary Fig. 1a–d and see Supplementary Methods for details). To build a defined gradient of water content in the agar plate, we used a four-quadrant agar test that was modified from a previous report^16^ (Methods, behavioural assay 2). The N2 worms also showed hydroavoidance in this assay (Fig. 1f,g, Supplementary Fig. 2a–c), confirming our observations in the WSA test.

To test the possibility that the worms react to the agar gradient, we used agarose and gelatin to make the wedge-shaped medium. The wild-type worms also exhibited hydroavoidance on the medium made of 2% agarose and 6% gelatin (Supplementary Fig. 1e); therefore, it is unlikely that the worms reacted to the agar gradient. Agar, even the high quality Bacto™ agar used in this study, is not a pure material and contains other chemicals. The contaminants may form a gradient in the wedge-shaped and four-quadrant agar plates that would make the worm prefer different areas of the plates, particularly the possible attractive chemicals that would attract the worms to the drier areas. To test the possible impact of contaminating inorganics on hydrosensation and hydrotaxis, we added CuSO_4_ as a repellent and sodium acetate (NaAc) as a chemoattractant into the agar or agarose media. *C. elegans* is sensitive to the noxious Cu^2+^ ion. More than 90% of the worm population did not remain in an area containing 100 μM Cu^2+^, and 30–40% of the worms escaped an area containing 10 μM Cu^2+^ ion^17^. The addition of 50 μM or 100 μM CuSO_4_ to the 2% wedge-shaped agar plates would create a gradient of Cu^2+^ to generate avoidance behaviour in worms. However, our test results showed that the addition of 50 μM or 100 μM CuSO_4_ did not affect worm hydrotaxis in the WSA assay (Supplementary Fig. 1f), suggesting that a gradient of possible repellents in agar did not interfere with the worms’ hydrotaxis in our tests. Based on the information in the agar manual and our ICP-MS (inductively coupled plasma mass spectrometry) and HPLC (high performance liquid chromatography) measurements of the agar and agarose wedge-shaped plates, the contamination with Na^+^ ions may be non-negligible. In the wedge-shaped agar plates, the levels of contaminating Na^+^ in the regions of >4.0 cm and <0.5 cm were 5.14 mM and 12.55 mM, respectively. In the wedge-shaped agarose plate, the Na^+^ content was 1.77 mM and 2.41 mM in the area of >4.0 cm and <0.5 cm, respectively. The concentrations of other ions, such as Ca^2+^, Mg^2+^, K^+^, Fe and SO_4_^2−^, are lower than 0.5 mM or undetectable. The worms are sensitive to the attractive Na^+^ ion^18^. Our results from the four-quadrant agarose test, in which different concentrations of the Na^+^ ion were added into two opposite quadrants (see Methods behavioural assay 2 for detail), showed that the wild-type animals were able to distinguish the difference in Na^+^ concentrations between 10 mM and 20 mM, and even between 0 mM and 1 mM, in the 2–2% agarose four-quadrant assay (Supplementary Fig. 2d,e). These results imply that the Na^+^ gradient may interfere with the worms’ hydrotaxis. To examine the possible impact of Na^+^ contamination on the worms’ preference for environmental water content (hydropreference), we used four-quadrant agarose medium with equal concentrations of Na^+^ (Methods, behavioural assay 3). Our results showed that the wild-type N2 worms also displayed an obvious preference for the drier areas of the agarose plates (Fig. 1h,i). In addition, we used a simple method to generate a 6–2% agarose plate with an equal amount of soluble chemicals, taking advantage of diffusion^19^ (Supplementary Fig. 3a,c). In this assay, the wild-type animals also showed a preference for the drier area, regardless of the presence of food (Supplementary Fig. 3b and d). These tests demonstrate that *C. elegans* can sense the water gradient in the substrate and show a preference for drier areas in the agar and agarose plates.

Furthermore, we used a wet-drop test and a dry-drop test, as previously described^20^, to examine the response of individual worms to water droplets and to a local increase in water content (Methods, behavioural assay 4). A drop (a few hundred nanolitres) of pure water or phosphate-buffered saline (PBS, osmolarity, 285 mOsmM) was delivered via a glass micropipette in front of a moving worm. As the worm moved forward, it would encounter either the droplet (wet-drop test) or the local moistened place (dry-drop test). N2 worms showed avoidance in both the dry-drop and wet-drop tests on either 2% agar plates or NGM plates. The presence or absence of food (*E. coli* OP50) had no significant effect on the worms’ avoidance of the water drops (Fig. 1j,k, Supplementary Movies 1 and 2). However, the worms rarely responded to PBS (Supplementary Movies 3 and 4, Supplementary Fig. 4). This observation suggested that the worms responded to the hypo-osmotic water drops or to a local increase in the water content, and the aversive behaviour was not evoked by mechanical stimulation. Together, our tests demonstrate that the worms can sense the water gradient in their culture substrate as well as sense hypo-osmotic water drops and can display hydroavoidance.

### The cGMP signalling pathway plays a major role in hydrosensation

**Figure 2:**
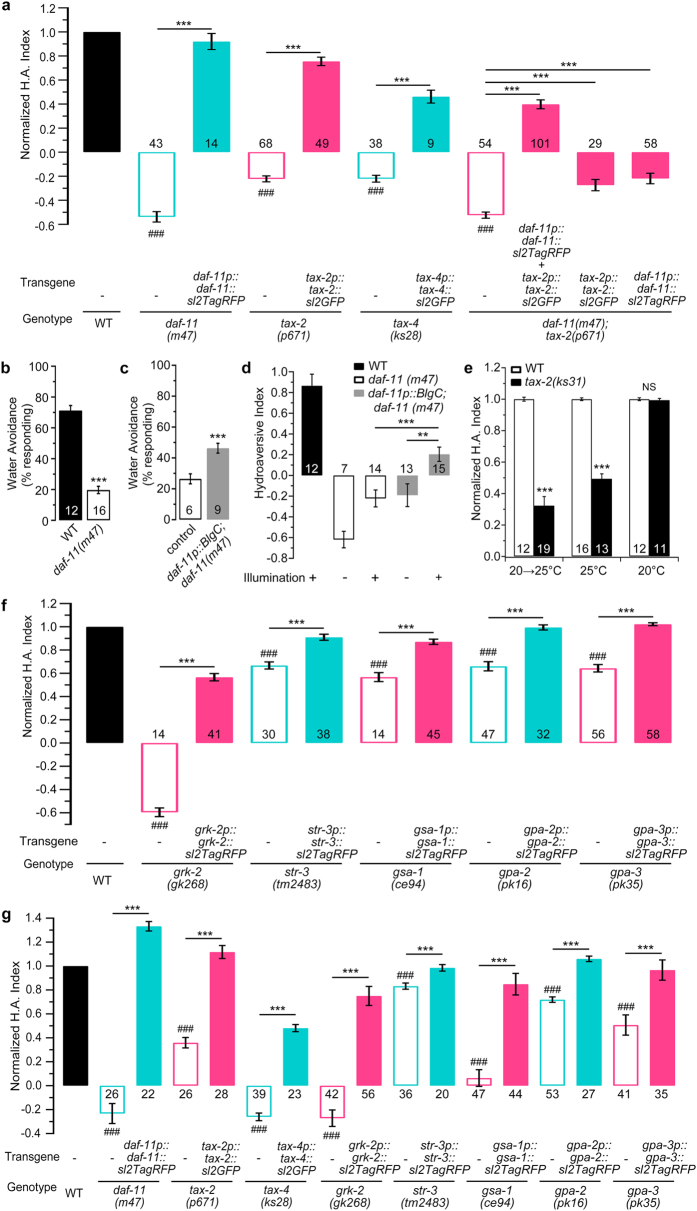
cGMP – TAX-2/TAX-4, G-protein-coupled receptor (GPCR) and G-protein signalling is involved in hydrosensation. (**a**) The normalised H.A. indexes of the *daf-11(m47)*, *tax-2(p671*), *tax-4(ks28)* and genetically rescued worms in the WSA assay. (**b**) Avoidance ratio of *daf-11(m47)* in the water drop test. (**c,d**) Photo-activation of BlgC in *daf-11*-expressing neurons rescued the hydrotaxis defects in *daf-11(m47)* mutants in the drop test (**c**) and the WSA assay (**d**). (**e**) The normalised H.A. indexes of the *tax-2(ks31)* mutants treated with a temperature shift for 24 h at young adulthood after being raised at the permissive or restrictive temperature in the WSA assay. (**f**) The normalised H.A. indexes of the GPCR and G-protein mutants and worms rescued with full-length genomic DNA in the WSA assay. (**g**) The normalised H.A. indexes of the *daf-11*, *tax-2*, *tax-4*, *grk-2*, *str-3*, *gpa-2*, and *gpa-3* mutants and genetically rescued worms in the four-quadrant agarose (6%-2%) assay with equal concentrations of Na^+^. The data for each genotype are normalised to the corresponding wild-type N2 control and are shown as only one bar. The number of independent tests for each genotype is indicated on the bar. Statistics: ^###^*P* ≤ 0.001 compared with each N2 control; ns, not significant; **P ≤ 0.01 and ***P ≤ 0.001 compared as indicated (Student’s *t*-test or Mann-Whitney Rank Sum test, depending on the normality of the data distribution). The error bars indicate the SEM. WT, wild-type; H.A., hydroaversive.

To identify the genes that may be involved in hydrosensation, we used reverse genetics to screen for the genes that function in chemosensation signalling and those encoding the channels associated with mechanosensation and water transport. Because of its greater feasibility, we used the WSA assay as the primary test method, and other test paradigms were used to verify the results of the WSA assay. With the exception of certain *mec* (mechanosensory abnormality) mutants, all of the water channel mutants tested (Supplementary Fig. 5a) and a large proportion of the *mec* mutants (Supplementary Fig. 5c) showed no significant differences in hydropreference compared to the wild-type worms. Fortunately, by using a reverse genetic screen, we identified that the *daf-11(m47)* mutants exhibited severe defects in hydropreference, as assessed by the WSA (Fig. 2a), four-quadrant agarose/agar (Fig. 2g and Supplementary Fig. 6a), long-term diffusion assay (Supplementary Fig. 3) and drop tests (Fig. 2b and Supplementary Fig. 6b); these defects were fully rescued in each assay by the extrachromosomal expression of the full-length *daf-11* genomic DNA driven by its own promoter. *daf-11* encodes a transmembrane guanylate cyclase that is reported to be required for axon formation, chemotaxis and the prevention of dauer formation^21^,^22^,^23^. Cyclic nucleotide-gated channels (CNGs) act as effectors of cGMP in chemosensation, oxygen sensing and photo-transduction in *C. elegans*^18^,^24^,^25^,^26^. Our genetic screen identified the cGMP-gated TAX-2/TAX-4 channel (Fig. 2a), but likely not other CNG channels (Supplementary Fig. 5b), as involved in hydropreference. To validate the function of cGMP in hydrotaxis, we optogenetically increased the cytosolic cGMP levels using a blue light-activated guanylate cyclase, BlgC^27^, which was expressed in the *daf-11* expressing neurons. Indeed, the artificial increase in the intracellular cGMP levels remarkably relieved the water avoidance (hydroaversive) defect in the *daf-11(m47)* mutants in the drop test (Fig. 2c) and the WSA assay (Fig. 2d). The DAF-11-TAX-2/TAX-4 signalling pathway regulates sensory axon^22^ and cilium growth (Supplementary Fig. 7a–d). To corroborate the post-developmental function of this signalling pathway in hydrosensation, we examined the hydroavoidance of temperature-sensitive *tax-2(ks31)* mutants^22^. When raised at the permissive temperature (20 °C), the mutants did not exhibit defects in ASI cilium structure or hydrotaxis (Fig. 2e, Supplementary Fig. 7e–g). Interestingly, when the mutants were allowed to develop into the young adult stage at 20 °C and then shifted to the restrictive temperature (25 °C) for 24 hours, the worms exhibited a hydrotaxis defect (Fig. 2e) similar to that of the animals raised at 25 °C, even though the temperature-shifted worms developed cilia with normal structure in ASI neurons (Supplementary Fig. 7e and f). Even a 12-hour temperature shift resulted in a hydroaversive effect similar to that of the 24-hour treatment (Supplementary Fig. 7g). These results demonstrate that DAF-11 signalling plays a role in adult hydrosensation and hydrotaxis. We next examined *tax-2(p671);daf-11(m47)* double mutants to confirm the upstream and downstream relationships in the signalling pathway. The double mutants displayed a hydroaversive defect similar to that of the *daf-11(m47)* mutants, whose defect was more severe than that of the *tax-2(p671)* mutants. Co-expression of *daf-11* and *tax-2*, which were driven by their own promoters, rescued the defect in the double mutants, but the separate expression of either *daf-11* or *tax-2* did not (Fig. 2a). Furthermore, a temporary light-induced increase in the cytosolic cGMP levels in the *daf-11* expressing neurons did not rescue the *tax-2(p671)* mutant (Supplementary Fig. 6c). These data demonstrate that *daf-11* is upstream of *tax-2* in the signalling cascade for hydrosensation. DAF-11 signalling is involved in chemosensation^18^, and the hydropreference defect in the mutants is likely caused by a defect in sensing the soluble chemicals in the substrate of the medium. To test this possibility, we used 6%–2% four-quadrant agarose plates with equal concentrations of Na^+^ and similar concentrations of soluble chemicals that were dispersed by long-term diffusion (for over 12 hours). Our results (Fig. 2g and Supplementary Fig. 3b and d) showed that the mutants and the genetically rescued worms displayed hydropreferences similar to those revealed by the WSA test (Fig. 2a) and that the hypothesised chemosensory defect of the *daf-11*, *tax-2* and *tax-4* mutants did not reverse the hydrotaxis defects in these worms. Here, we demonstrated that DAF-11 – cGMP – TAX-2/TAX-4 signalling functions autonomously in adult hydrosensation in addition to its role in cilium development.

### GPCR signalling functions in hydrosensation

Receptor-like guanylate cyclases (RGCs) are classified into ligand-dependent and ligand-independent classes. In olfaction in *C. elegans*, DAF-11 acts downstream of G protein signalling in a ligand-independent manner^18^,^21^. We therefore addressed whether G-protein-coupled receptors (GPCRs) and G-proteins play roles in hydrosensation signal transduction. Our WSA test results showed that mutations in GPCRs, GPCR kinases, G-proteins and their regulatory proteins (Fig. 2f, Supplementary Fig. 8) caused hydroaversive defects. Our data from the assay using the four-quadrant agarose plates with equal concentrations of Na^+^ (Fig. 2g) supported the hypothesis that the observed defects of hydropreference in the mutants and the genetically rescued worms in the WSA assay were not caused by defects in chemosensation of the soluble chemicals in the agar or agarose substrate. We screened all of the α, β, and γ G-protein subunits. However, we found no significant defects when knocking out any single β or γ subunit, likely because they are usually functionally redundant. The rescue effects of the GPCR *str-3*, the GPCR kinase *grk-2,* and the G-protein subunits *gsa-1*, *gpa-2* and *gpa-3* (Fig. 2f,g) strongly suggest that GPCRs and G-proteins play important roles in hydrosensory information transduction. The loss-of-function *grk-2* mutation results in reduced GPCR signal transduction^28^. Notably, the *grk-2(gk268)* mutants have a very severe hydroaversive defect, similar to the *daf-11(m47)* worms, suggesting that GPCR signalling is involved in hydrosensation and may play an essential role in the transmembrane transduction of hydrosensory information. Together, our data demonstrate that GPCR signalling and cGMP signalling play a dominant role in water sensing and hydrotaxis in the worms.

### Identification of candidate water-sensing cells

**Figure 3:**
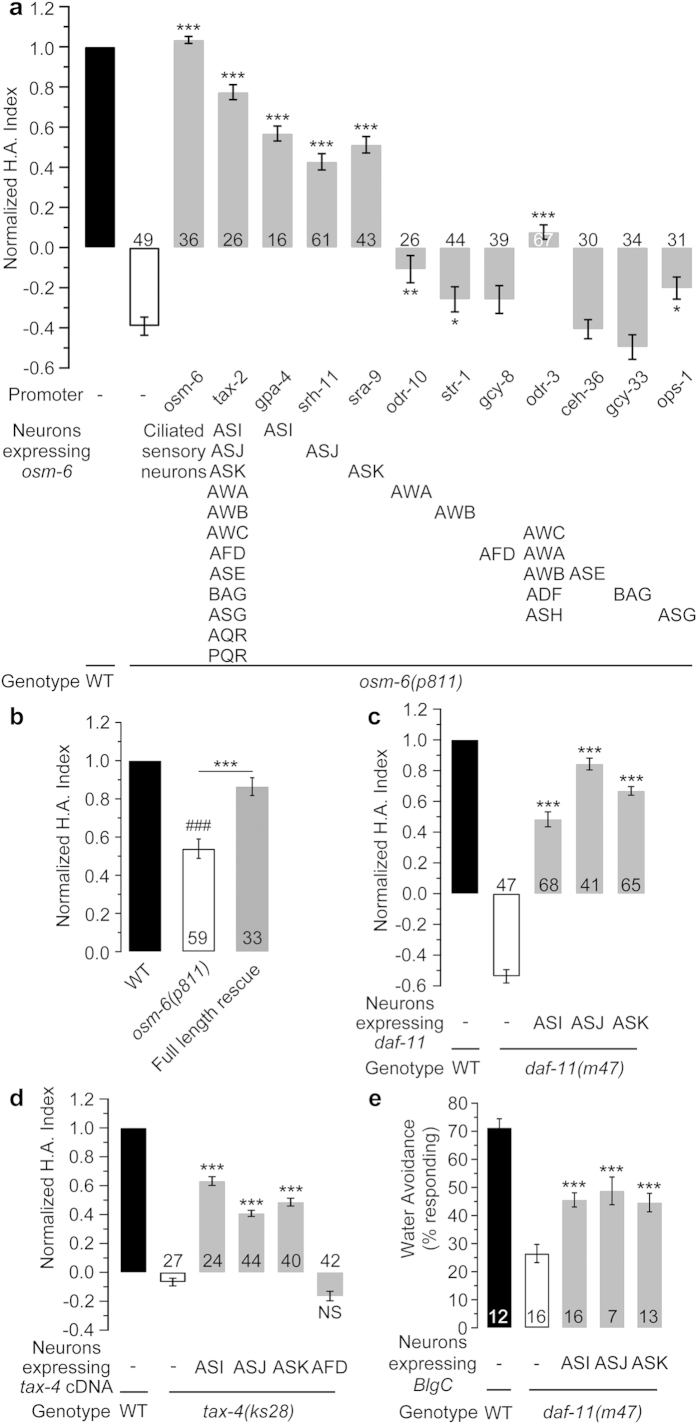
Identification of the hydrosensory receptor neurons. (**a**) The normalised H.A. index of the *osm-6(p811)* mutants and neuron-specific rescued worms, as indicated by the WSA assay. (**b**) The normalised H.A. index of the *osm-6(p811)* mutants and worms rescued with the full-length *osm-6* genomic DNA, as indicated by the four-quadrant agarose assay with equal concentrations of Na^+^. (**c**) The normalised H.A. indexes of the *daf-11(m47)* mutants and worms that were specifically rescued with the full-length *daf-11* genomic DNA in the ASI, ASJ and ASK neurons, as tested by the WSA assay. (**d**) The normalised H.A. indexes of the *tax-4(ks28)* mutants and worms that were specifically rescued with the *tax-4* cDNA in the ASI, ASJ, ASK, and AFD neurons, as tested by the WSA assay. (**e**) The artificial increase in the cytosolic cGMP levels by photo-activation of BlgC expressed specifically in the ASI, ASJ and ASK neurons remarkably rescued the hydropreference defect of the *daf-11(m47)* mutants, as assayed by the drop test. The data for each genotype are normalised to the corresponding wild-type N2 control, which is shown as only one bar. The number of independent tests for each genotype is indicated on the bar. Statistics: *P ≤ 0.05, **P ≤ 0.01 and ***P ≤ 0.001 compared to each N2 control (Student’s *t*-test or Mann-Whitney Rank Sum test, depending on the normality of the data distribution). The error bars indicate the SEM. WT, wild-type; H.A., hydroaversive.

The primary cilia are sensory organelles in both vertebrates and invertebrates, and cGMP signalling is fundamental for ciliary structure and function in eukaryotes^24^,^29^,^30^. Therefore, we examined mutants of genes that are essential for intraflagellar transport and ciliary structure. Indeed, our data showed that most of these mutants exhibited serious defects in hydropreference, as tested by the WSA assay (Supplementary Fig. 9a). These data suggest that the ciliated sensory neurons are most likely the hydroreceptor cells. *osm-6* is known to be involved in the development of all ciliated chemosensory and mechanosensory neurons^31^, and *osm-6(p811)* mutants showed a severe cilium defect (Supplementary Fig. 9b,c). Moreover, the defective phenotype of the *osm-6(p811)* mutants was also confirmed by the four-quadrant agarose assay (Fig. 3b) and long-term diffusion assay (Supplementary Fig. 3b and d). Therefore, we performed a series of rescue experiments using *osm-6* genomic DNA in the *osm-6(p811)* mutants to identify the candidate water-sensing cells. The extrachromosomal expression of *osm-6* DNA driven by its own promoter or that of *tax-2* fully rescued the hydropreference in the *osm-6* mutants, as assayed by the WSA test (Fig. 3a), the four-quadrant agarose assay (Fig. 3b), the four-quadrant agar assay (Supplementary Fig. 10a) and the drop test (Supplementary Fig. 10b). Interestingly, the specific ectopic expression of the gene in the either the ASI, ASK or ASJ ciliated sensory neurons also efficiently restored the hydropreference in the mutants (Fig. 3a). The ASI, ASJ and ASK ciliated sensory neurons possess DAF-11 – TAX-2/TAX-4 signalling. We then used *daf-11* and *tax-4* rescues to confirm the above results. Specific and ectopic expression of the *daf-11* genomic DNA in the pairs of ASI, ASK or ASJ neurons was sufficient to yield a significant rescue effect in the WSA test (Fig. 3c). Likewise, the *tax-4* mutants could also be rescued by *tax-4* cDNA expression specifically in the ASI, ASJ, or ASK neurons but not in the AFD neurons (Fig. 3d). Furthermore, the optogenetic increase in the cytosolic cGMP levels by photo-activation of BlgC expressed specifically in any of these three neuron pairs also significantly rescued the hydroaversive defects in the *daf-11(m47)* mutants (Fig. 3e).

### The ASJ, ASK and ASI neurons are sensitive to the switches among buffer, water and air

**Figure 4:**
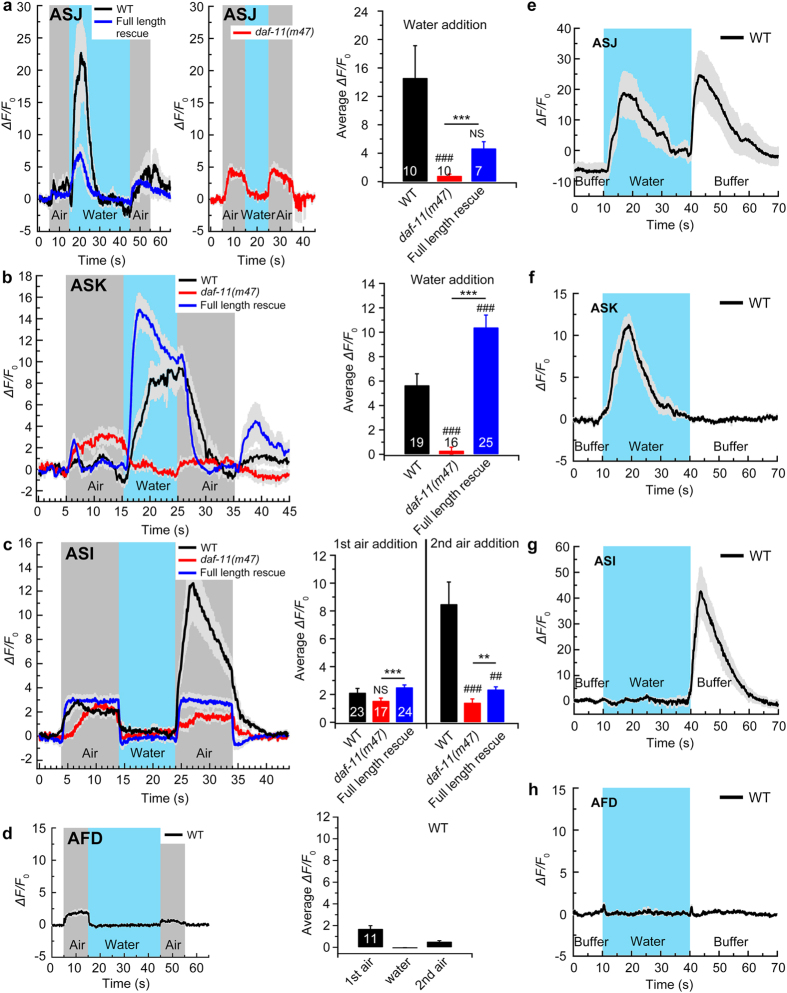
The ASI, ASJ and ASK neurons sense switching between buffer, air and water. (**a–d**) Cytosolic Ca^2+^ signalling in the soma in response to switches between the M13 buffer, air and ultra-pure water, as indicated in the ASJ (**a**) ASK (**b**) ASI (**c**) and AFD (**d**) neurons. Before and after air and water application, the worms were in the M13 buffer solution. The dark grey and blue shading indicate the challenge of air and water flow, respectively. A summation of the average fluorescence responses in the ASJ, ASK, ASI and AFD neurons is shown in each right-hand panel. The fluorescence signal within the initial 10 s was calculated as the average value for the response of the ASJ neurons in wild-type and rescued animals (**a**) to water stimulation. (**e–h**) Cytosolic Ca^2+^ signals in the soma in response to the switch between the M13 buffer and ultra-pure water in the ASJ (n = 8), ASK (n = 10), ASI (n = 10) and AFD (n = 16) neurons. The blue shading indicates the addition of water. The data are shown as the means ± SEM, as indicated by the solid traces in colours indicated and light grey, respectively. The full-length rescue indicates the transgenes expressing full-length *daf-11* genomic DNA. The number of independent tests is indicated for each genotype on the bar. Statistics: ^###^*P* ≤ 0.001 compared to each N2 control, ***P ≤ 0.001 compared as indicated (Student’s *t*-test or Mann-Whitney Rank Sum test, depending on the normality of the data distribution). The error bars indicate the SEM. WT, wild-type.

To further examine the mechanism of hydrosensation, we recorded the changes in the cytosolic calcium levels in the somas of the ASI, ASJ and ASK neurons using genetically encoded calcium indicators (R-GECO1.0, G-GECO1.1 and G-CaMP2)^32^,^33^ combined with microfluidic chips^34^. In wild-type animals, these three pairs of neurons responded to buffer-air, air-water or water-air switches with different kinetics. The ASJ (Fig. 4a) and ASK (Fig. 4b) neurons displayed rapid increases in the calcium levels when switched to an air-water flow or the addition of water, whereas the ASI neurons exhibited a rapid increase in the calcium levels in response to water-air stimulation or the removal of water (Fig. 4c). The calcium responses in all three types of neurons were almost absent in the *daf-11(m47)* mutants and were remarkably rescued by extrachromosomal expression of the full-length *daf-11* genomic DNA (Fig. 4a–c). Interestingly, the ASJ, ASK and ASI neurons showed calcium responses to the buffer-water switch (Fig. 4e–g) that were similar to those to the air-water switch (Fig. 4a–c), hinting that the calcium signals were mainly caused by the addition or removal of water. AFD neurons displayed no Ca^2+^ response to the switch between buffer and water (Fig. 4h) but exhibited weak Ca^2+^ signals in response to the switch to buffer-air, similar to the responses of the ASI neurons. The AFD and ASI neurons are known to be thermosensitive^13^,^35^,^36^,^37^, and these calcium signals may have been caused by a subtle temperature change (Fig. 4d). However, further studies are needed to support this hypothesis. All of these results suggested that the ASK, ASJ and ASI neurons sense the addition or removal of water and that they are water-sensing neurons.

## Discussion

The external or internal water content may ultimately change cellular osmolality, volume and stress^10^, and the environmental water content may affect the temperature in animals’ habitats. Water sensing may be a complex process involving chemo-, mechano-, osmo- and even thermosensation pathways. The mechanosensitive channels water witch and nanchung of the transient receptor potential channel family^2^ and the osmosensitive ion channel PPK28^1^,^11^ of the degenerin/epithelial sodium channel family are involved in humidity sensing in adult *Drosophila*. Our results show that some *mec* genes, which function in mechanosensation^38^, may play minor roles in sensing the water content in the substrate (Supplementary Fig. 5c). These studies support the idea that water sensing is a complex process. cGMP signalling plays crucial roles in cilium development, growth and function^24^. In *C. elegans*, the expression of the components of this signalling pathway is restricted to the ciliated sensory neurons, with a few exceptions^24^. The cGMP signalling pathway plays essential roles in the chemosensation of soluble and volatile compounds^18^, oxygen^39^ and CO_2_^40^, as well as in photosensation^26^. This pathway is also involved in locomotion on medium surfaces and in swimming^41^ and in long-term physiological processes, including satiety, lifespan, lethargy, innate immunity and development^42^,^43^,^44^. The cGMP pathway is also important for the development and growth of plant roots^45^,^46^,^47^. DAF-11 is a receptor-like guanylate cyclase and acts downstream of G-protein-coupled receptors. *daf-11* is involved in pheromone sensation, dauer formation^48^,^49^,^50^, satiety quiescence^51^, chemosensation, chemotaxis^21^,^23^,^52^,^53^, photosensing^26^, and food-odour sensing and preference^54^. The hydrosensory function that we discovered adds a new and important aspect to the cGMP signalling pathway.

**Figure 5:**
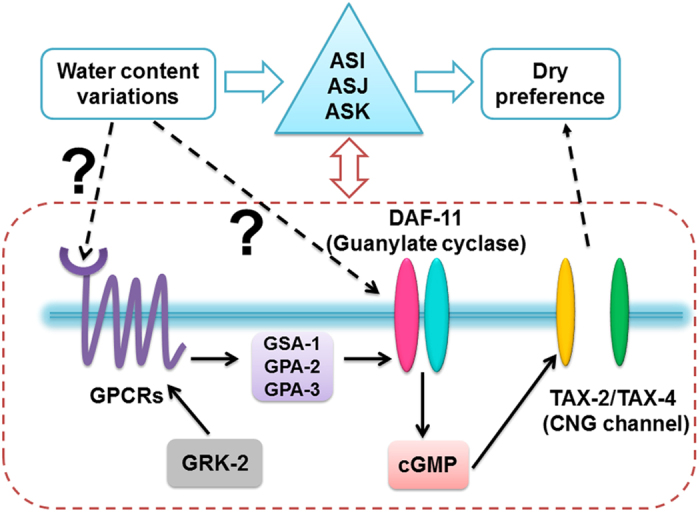
Working model of hydrosensory information transduction. Hypothesised model of the hydrosensation signalling pathway, i.e., a transmembrane hydrosensory information transduction pathway. The receptor-like guanylate cyclase DAF-11 may be activated directly or indirectly via G proteins through GPCR signalling, resulting in an increase in cytosolic cGMP levels that opens the CNG channels.

Water in the substrate provides an essential environment for the locomotion and survival of *C. elegans*^55^. Adult worms cannot survive for 24 hours without a moist agar substrate, even at a high RH of 98%^56^. It is essential for such animals to develop water sensing and hydrotaxis mechanisms. Our results show that *C. elegans* can sense the water in the agar substrate and exhibit a behavioural preference for the drier areas in the agar plates, in addition to their previously reported humidity-sensing capabilities^13^. Starvation-primed worms display a robust preference for drier air through mechanosensation and thermosensation^13^. The differences in the water content in agar medium may create a subtle temperature difference in or near the medium surface. Therefore, hygrosensation may play a role in sensing the water content in an agar substrate. Our data show that starvation has a weak impact on hydrotaxis (Fig. 1k and Supplementary Fig. 3), and the ASI and AFD neurons, which are known thermosensitive neurons^13^,^35^,^36^,^37^, display weak responses to the buffer-air switch (Fig. 4), which may be caused by a subtle temperature change. These results suggest that thermosensation in the ASI and AFD neurons may play a minor role in hydrosensation. However, our overall data strongly suggest that the GPCR – G-protein – DAF-11 – cGMP – TAX-2/TAX-4 signalling pathway mediates *C. elegans* hydrosensation, as shown in Fig. 5. In this sensory information transduction pathway, what is (are) molecular sensor(s), GPCR, DAF-11 or both of them? DAF-11 is regarded to act downstream of G protein signalling in a ligand-independent manner^20^,^23^. One possibility is that GPCRs may directly or indirectly detect the extracellular water content or its changes and then activate the guanylate cyclase DAF-11 via G protein-mediated transduction, resulting in increased cytosolic cGMP levels that open the CNG channels. Our data show that mutations of the genes encoding the GPCR STR-3, the GPCR kinase GRK-2, and the G-protein subunits GSA-1, GPA-2 and GPA-3 cause hydroaversive defects (Fig. 2f,g, Supplementary Fig. 8b). In particular, the *grk-2* mutation has a phenotype similar to that in the *daf-11* mutants. These results suggest that GPCRs may be water sensors. However, this hypothesis requires direct supporting evidence. Because guanylate cyclases are involved in sensing O_2_^39^, CO_2_^40^, NO^57^ and even cool temperature^58^, and atrial natriuretic peptide receptors are involved in the homeostasis of water and sodium^59^, the possibility that DAF-11 senses the water content and that GPCR signalling only acts as a regulator is attractive. The most severe hydrotaxis defect was observed in the *daf-11(m47)* mutants, which suggests that DAF-11/cGMP signalling has a pivotal function in sensory information transduction. However, direct evidence is needed to support this hypothesis.

Two types of hygroreceptors exist in insects: one responds to an increase (moist receptor) and the other to a reduction (dry receptor) in humidity^2^,^60^. In *Drosophila*, *water witch* (*wtrw*) and *nanchung* (*nan*) are required to detect moist and dry air, respectively^2^. In animals, the use of two or more information inputs is more adaptive than using only one information input. Our data show that the *daf-11*, *tax-2*, *tax-4*, and *grk-2* mutants not only fail to avoid the wetter areas but appear to prefer moist places. The results imply that these genes may be involved in a process that drives the worms to the dry areas. Meanwhile, the success in raising the hydroaversive index (H.A.) from −0.4 to zero for the rescue of *osm-6* driven by *odr-3* promoter in odour, ADF and ASH neurons implies that counterpart to this process may take part in hydrosensation (Fig. 3a). However, in the wild-type animals, the process that drives the animals to the dry areas seems to prevail. To fully understand hydrosensation and hydrotaxis, the neuronal circuit must be dissected, and the mechanism, *i.e.*, neuronal information transduction, integration and analysis, in the central nervous system must be identified.

Hydrosensation is important not only for the detection of external water sources by peripheral neurons but also essential for osmosensation by the central nervous system and for systemic osmoregulation^10^. Our present work provides a molecular basis for recognising *C. elegans* hydrosensation and a framework for examining water sensing in the peripheral and central nervous systems in other animals, including humans.

## Methods

### Behavioural Assay 1: The cylindrical wedge-shaped agar (WSA) assay paradigm

For this assay, 12 ml of hot 2% agar solution (w/v, in ultra-pure water) was poured into a 9-cm-diameter Petri dish on a shelf with a 15 degree inclination and allowed to solidify and dry at 20 °C and approximately 40% relative humidity for approximately 40 min without a cover. After drying, a visible edge was formed at the front of the thin area of the agar plate. Then, the wedge-shaped agar plate was closed with a lid and used on the same day. The tested worms were washed 3 times with phosphate-buffered saline (osmolarity, 285 mOsmM) and pipetted in a small volume (approximately 10 μl) onto an agar plate along a line at a 1.0 cm distance from the edge of the thin area. The excess liquid was removed with a tissue, and the plate was closed with a lid. In total, 100 to 200 worms were used for each test. The test plates were placed in the dark at 20 °C for 100 min on a shelf with 15 degree inclination to make the agar plane horizontal. The hydroaversive (H.A.) index is calculated as H.A. = (Worms _<0.5_ _cm_ – Worms _>1_ _cm_)/(Worms _<0.5_ _cm_ + Worms _>1_ _cm_). Unless otherwise indicated, the data for all genotypes are normalised to their own N2 control and are shown as only one bar in each graph. For the adult temperature shift experiments, the animals were allowed to develop into the young adult stage at 20 °C, then shifted to 25 °C for 12 h or 24 h, and were subsequently used for the hydrotaxis assays and imaging. For the fixed-temperature experiments, the worms were allowed to develop into the adult stage at the permissive (20 °C) or restrictive (25 °C) temperatures. Identically treated wild-type N2 animals served as the controls.

### Behavioural Assay 2: The four-quadrant agar test paradigm

To test the worms’ hydropreference in the four-quadrant agar plates, three sets of four-quadrant agar plates, 2–4%, 2–6%, and 2–2% (control), were made, with some of the modifications used in the Cu^2+^ -avoidance assays^33^,^53^. Approximately 22 ml of a hot 2%, 4% or 6% agar solution (w/v in ultra-pure water only) was poured into a 9-cm Petri dish and allowed to solidify at room temperature for ~10 min; then, the agar from opposite quadrants and the central area was removed and replaced with a 2% agar solution. The plates were left open to solidify and dry for an additional ~60 min. Then, the plates were closed and used on the same day. The washed animals were placed in the centre of the four-quadrant agar plate and scored under a stereomicroscope at 100 min. A hydroaversive index (H.A. Index = (A – C)/(A + C)) was calculated, where A is the number of worms over the area that had been filled with 2%, 4% or 6% agar, and C is the number of worms over the area that was filled with 2% agar. The assay plates remained unsealed during the entire procedure.

### Behavioural Assay 3: four-quadrant agarose test paradigm with equal concentrations of Na^+^

This paradigm used 2% and 6% agarose in a 100 mM sodium acetate (NaAc) solution, and extra NaAc was added into the 2% agarose to make the concentration of Na^+^ equal to that in the 6% agarose. A hydroaversive index (H.A. Index = (A – C)/(A + C)) was calculated, where A is the number of worms over the 6% agarose area and C is the number of worms over the subsequently poured 2% agarose area. The assay plates remained unsealed during the entire procedure.

### Behavioural Assay 4: Drop test

To examine the hydroavoidance of a single worm, the water drop test and dry-drop test were performed as previously described^17^,^34^. Approximately 30 worms were placed on an agar plate (2% in ultra-pure water) without a bacterial lawn that was prepared 2 hours before the experiment or on a normal NGM plate. After acclimation for 8 minutes, the worms were stimulated with a water drop. A microdrop (a few hundred nanolitres) of pure water or PBS was delivered via a glass micropipette in front of an animal exhibiting forward sinusoidal locomotion. When the nose of the worm touched the water drop (drop test) or the local area where a water drop was dripped onto the plate and absorbed into the agar (dry-drop test), the worm displayed a backward movement (Supplementary Movies S1 and S2). However, the worms displayed no response to the PBS drop in the drop and dry-drop tests (Supplementary Movies S3 and S4). The worms’ responses were observed and recorded under a Zeiss Discovery V8 stereomicroscope (Carl Zeiss MicroImaging GmbH, Göttingen, Germany). The image sequences were captured with an Andor DV885K EMCCD camera (Andor Technology plc, Springvale Business Park, Belfast, UK). The avoidance index represents the fraction of animals reversing for more than one-half of the worm’s length within 4 s among all of the worms in a test.

A full description of the materials and methods can be found in the Supplementary Materials and Methods.

## Additional Information

**How to cite this article**: Wang, W. *et al.* cGMP Signaling Mediates Water Sensation (Hydrosensation) and Hydrotaxis in *Caenorhabditis elegans.* *Sci. Rep.* **6**, 19779; doi: 10.1038/srep19779 (2016).

## Supplementary Material

Supplementary Information

Supplementary Movie 1

Supplementary Movie 2

Supplementary Movie 3

Supplementary Movie 4
