# Supplementary material for: cGMP Signalling Mediates Water Sensation (Hydrosensation) and Hydrotaxis in Caenorhabditis elegans
Source: Sci Rep. 2016 Feb 19;6:19779. doi: 10.1038/srep19779 (PMC4759535; doi:10.1038/srep19779)
Supplement: Supplementary Information [file srep19779-s1.pdf]

Supplementary Information

cGMP Signalling Mediates Water Sensation (Hydrosensation) and Hydrotaxis in *Caenorhabditis elegans*

Wei Wang*, Li-Wei Qin*, Tai-Hong Wu*, Chang-Li Ge, Ya-Qian Wu, Qiang Zhang, Yan-Xue Song, Yuan-Hua Chen, Ming-Hai Ge, Jing-Jing Wu, Hui Liu, Yao Xu, Chun-Ming Su, Lan-Lan Li, Jing Tang, Zhao-Yu Li, and Zheng-Xing Wu

Key Laboratory of Molecular Biophysics of Ministry of Education, Institute of Biophysics and Biochemistry, and Department of Biophysics and Molecular Physiology, College of Life Science and Technology, Huazhong University of Science and Technology, Wuhan, 430074, P.R. China

*These authors contributed equally to this work. Correspondence and requests for materials should be addressed to Z.X.W. (email: ibbwuzx@mail.hust.edu.cn).

Supplementary Materials and Methods

Supplementary Figures

Supplementary Video Legends

Supplementary Table

Supplementary Materials and Methods

Strains. All strains were maintained and grown on nematode growth media (NGM) plates at 20 °C fed with *E. coli* strain OP50 according to the standard procedures[61], unless otherwise indicated. Strains were obtained from the CGC (http://www.cbs.umn.edu/CGC/) and the National Bio-Resources Project (http://www.shigen.nig.ac.jp/c.elegans/index.jsp). Double mutant animals were generated using the standard genetic techniques[61] and confirmed by PCR or sequencing. All transgenic strains were generated using the standard microinjection techniques[62]. Plasmids were injected at 50 - 70 ng μl^{-1} together with *vha-6p::GFP* (5 ng μl^{-1}), *myo-2p::mKate2* (5 ng μl^{-1}) or *lin-44p::GFP* (10 ng μl^{-1}) as a co-injection marker. The mutants and transgenic *Caenorhabditis elegans* strains used in this study are listed in Table S1.

Molecular Biology. Three-Fragment Multisite Gateway® (Invitrogen™, Thermo Fisher Scientific) and In-Fusion® (Clontech® Laboratories, Inc.,) technology was used to generate the constructs used in this study. Briefly, three entry clones comprising three PCR products (promoter, gene of interest, *sl2e::GFP*, *sl2e::TagRFP-t* or *3'UTR*) were recombined into the pDEST™ R4-R3 Vector II or a custom modified destination vector using LR recombination reactions to generate the expression plasmids. An *att*R4-*ccdB-CmR*-*att*R3 cassette flanked with *Kpn* I restriction sites (both ends) was amplified from pDEST™ R4-R3 Vector II and inserted into the vector pPD49.26 (a gift from A. Fire) to generate the modified destination vector. The genes *daf-11*, *tax-2*, *tax-4*, *grk-2*, *str-3*, *gsa-1*, *gpa-2*, *gpa-3*, *gpa-4*, *gpa-5*, *gpa-14* and *osm-6* were amplified from N2 wild-type genomic DNA. The *sra-6* cDNA was amplified by RT-PCR from *C. elegans* mixed stage RNA.

Promoters

All promoters were amplified from N2 wild-type genomic DNA. The sequences of the promoter ends are shown below:

tax-2p: CTCCTCGAGAAATAGAATTCAG, ATCGGAAAACTCCGGTTTTT

sra-6p: ACTGACTGGGCCGGCCCC, GGAGCAGCACAACTTAAA

odr-10p: TTCCCCCACGTCAGACACC, CTGTAAGGTATCTTAAT

str-2p: AGAGACAGGATACAACTGAT, TTTTTATGGATCACGAGTATTC

srd-1p: GATCAGAAAAACAGTAGCG, CTTGATGATAAACAATCAAGTG

ceh-36p: CTTTGCAAGGCTTCATTC, GCGGGGGCAGGCGAAGT

gcy-8p: GGGCGAATTGGGTACATTAG, TTTGATGTGGAAAAGGTAG

str-3p: TCCAACTCGGAATACTACATAC, TCCAACTCGGAATACTACATAC

srh-11p: CCGCTGAATTCCTGAACTTGTC, CATGTCAATCGAACTATTC

daf-11p: TGGGAATAAAATAACGACG, GGTCCCATAGTAATTCTAT

gpa-2p: GAAGTATATCACAAATGCTTTG, TTTCTGAAAAACAGATGTTTGT

gpa-3p: ATCGGAATAAATACCAAGCGTT, AATTGTTCTTTGGCTAATAATT

gpa-4p: TGCGACTTTCGATACGTAGGTC, TGTTGAAAAGTGTTCACAAAATGA

osm-6p: AATGGAAGACGAGAGACAGTTG, AGATGTATACTAATGAAGGTAATAG

gcy-33p: AAGAAAAGTGATCCATTAAGAA, GTTTCTGAAATTAGGGTGTCTC

gcy-5p: GCTTTTCAATTGACTCTTGCAA, ACTCTAGATTTTGTACTCACCC

grk-2p: CAAAACTGATTATAGTTTATTATTTC, TTTTGTTCTGCAAAATCGAATT

gsa-1p: GATATTTTAACAAAATTCTAGC, CATAAGGCGCATCTTGACGCGG

ops-1p: AAAAGATTTTTTAGGACATTGT, TCATTTTTAGAGTTTCTGGAAA

sra-9p: CTGCAACACATCATTTTTCGTGGATC, GAAATCTTGAAACTGAAAAATACAAAAG

str-1p: GAGTGAGAAGAATGTCACGACT, CATTAGTCAAATGATATGAAGTTTG

tax-4p: ACATGCCTGAGTTGGGATTTTG, CATTCTTGAAACATAATTAAAT

Water content measurement. The water content of agar was calculated by the formula: (wet weight – dry weight) / wet weight. A small slice of agar was taken from the behavioural plate and weighed as wet weight. The same slice of agar was weighed for dry weight after full drying by baking at 55 ℃ for 24h. For wedge-shaped agar, the agar slices were taken every 0.5 cm from area A (Fig. 1a).

Worm tracking and locomotion analysis. Briefly, 40 - 50 washed worms were dripped on a piece of uniform or wedge-like agar plate 2.0 cm × 2.5 cm in size, then were imaged under a Zeiss Discovery V8 stereomicroscope (Carl Zeiss MicroImaging

GmbH, Göttingen, Germany). Images were recorded at 1 frame per sec for 100 minutes with an Andor iXonEM+ DV-885K-CS0-#VP-500 EMCCD camera (Andor Technology plc., Springvale Business Park, Belfast, United Kingdom), which was controlled by Andor iQ2.2 software, and analysed with the Multi-Worm Tracker (MWT)[63] and custom-written scripts for MATLAB (The MathWorks, Natick, MA, USA).

The assay on 6 % - 2 % agarose plate with long-term diffusion. The agarose plate was made of two layers of agarose. Each layer consisted of a semicircle of 6 % agarose and a semicircle of 2 % agarose. In the vertical direction, the two layers were made of 2 % (top) – 6 % (bottom) agarose and 6 % (top) – 2 % (bottom) agarose. Briefly, eight millilitres of hot 6 % agarose sol (W/V, in ultra-pure water) was poured into a 6 cm (in diameter) petri dish. When the 6 % agarose solidified, a semicircle was removed and refilled with 4 ml 2 % agarose sol. After the 2 % agarose cooled, another 8 ml of 6 % agarose sol was poured on the first layer. After cooling, the semicircle of the upper layer of 6 % agarose plate on top of 6 % agarose was carefully removed and refilled with 4 ml 2 % agarose sol. After the agarose solidified, the plates were sealed with parafilm and stored at 20 ℃ over 12h for diffusion. Then, the top layer of agarose was removed, and they were baked 20 min at 37 ℃. For the plates with food, 3 × OP50 was smeared on the surface of the agarose before baking. Washed animals were placed in the centre of a 6 % - 2 % agarose plate and scored under a stereomicroscope at 40 min. The hydroaversive index (H.A. Index = $(N_{6\%} – N_{2\%}) / (N_{6\%} + N_{2\%})$) was calculated. The assay plates were not sealed during the whole procedure.

Optogenetic Manipulation of Intracellular cGMP by Photoactivation of BlgC and Behaviour Tests. To optogenetically manipulate intracellular cGMP levels in neurons, transgenes expressing the blue light activated guanylyl cyclase BlgC[27], driven under promoters of *daf-11*, *srh-11* (ASJ specific), *sra-9* (ASK specific) and *gpa-4* (ASI specific), were used and illuminated by blue light. Behaviour was assayed by the drop test and the wedge-shaped agar (WSA) tests. For the drop test, 40 – 50 washed

transgenics were dripped onto a 2 % pure agar plate, and tested for their hydroavoidance under a Zeiss fluorescent stereomicroscope after acclimation for 10 minutes on the test plate. The worms were illuminated by continuous blue laser light (450 nm, ~ 10 mW / cm^2) during the whole procedure. Identically treated *daf-11(m47)* or *tax-2(p671)* mutants were used as controls. For the WSA testing, the transgenics were illuminated with a round blue light with diameter of 9.5 cm sourced from a 100 W LED array (460 – 470 nm, ~ 0.5 mW/cm^2) constructed in an LED light source (InBio Life Science Instrument Co. LTD, Wuhan, China), and scored at 40 min.

Calcium Imaging. Neuronal calcium responses in soma were measured by detecting changes in the fluorescence intensity of genetically encoded Ca^{2+} indicators R-GECO 1.0 and G-GECO 1.1 which are sensitive and show rapid kinetics and weak photo-bleaching[33], or G-CaMP 2.0[32]. For Ca^{2+} imaging of ASI, ASJ, ASK and AFD neurons, wild-type N2 animals and the transgenics ZXW960, ZXW973, ZXW976 and ZXW979 were used, in which R-GECO 1.0 expression was driven by the promoters of *srd-1*, *srh-11*, *sra-9* and *gcy-8*, respectively. For ASI, ASJ, and ASK Ca^{2+} imaging in *daf-11* mutants, R-GECO 1.0 was expressed under direction of the *gpa-4* promoter (transgene ZXW961), *srh-11* promoter (transgene ZXW974) and *sra-9* promoter (transgene ZXW977). For the ASI and ASJ Ca^{2+} imaging in *daf-11* rescued worms, G-GECO 1.1 was expressed under the *gpa-4* promoter (transgene ZXW962) and *srh-11* promoter (transgene ZXW975). For the ASK Ca^{2+} imaging in *daf-11* rescued worms, G-CaMP 2.0 was expressed under direction of the *sra-9* promoter (transgene ZXW978). A home-made microfluidic device was used for calcium imaging as previously described[34,64,65]. Briefly, a worm was immobilized by trapping in a micro-channel of the microfluidic chip, and the head of the worm was exposed to water or air flow. Laminar flow controlled through two alternatively on-off laminar streams was used to control the delivery of stimuli. The flows of air, ultra-pure water and buffer were delivered using a programmable automatic drug-feeding apparatus (MPS-2, InBio Life Science Instrument Co. LTD). R-GECO 1.0 was excited by 525 – 530 nm light emitted by an Osram Diamond Dragon LTW5AP light-emitting diode (LED) model (Osram, Munich,

Germany) constructed in a multi-LED light source (MLS102, InBio Life Science Instrument Co. LTD), and filtered with a Semrock FF01-593/40-25 emission filter (Semrock, Inc., Rochester, NY, USA), under an Olympus IX-70 inverted microscope (Olympus, Tokyo, Japan) equipped with a 40 × objective lens (numerical aperture (NA) = 1.3, Zeiss, Germany). G-GECO1.1 was excited by a 460 – 470 nm light emitted by an Osram Diamond Dragon LBW5AP LED model constructed in the MLS102 multi-LED light source and filtered with a Semrock 520/35 emission filter. Fluorescence images were captured with an Andor iXon^{EM}+ DU885K EMCCD camera with 100 ms exposure time and 256 × 256 pixels at 10 frames per second. The averaged fluorescence intensity of the region of interest (ROI) of the soma was captured and analysed by use of Image-Pro Plus 6.0 (Media Cybernetics Inc., Rockville, MD, USA), and photo bleaching was corrected by using a custom-written MATLAB script. The average fluorescence intensity within the initial 4 s before stimulation was taken as the basal signal F_0. The percent change of fluorescence intensity relative to the initial intensity F_0, $\Delta F = (F - F_0) / F_0 \times 100$ %, was plotted as a function of time for all curves. The mean values of Ca^{2+} signals and the SEM were plotted in various colours as indicated and in light grey, respectively, by use of IGOR Pro 6.10 (Wavemetrics, Portland, OR, USA).

References

61 Brenner, S. The genetics of *Caenorhabditis elegans*. *Genetics* **77**, 71-94, (1974).

62 Mello, C. C., Kramer, J. M., Stinchcomb, D. & Ambros, V. Efficient gene transfer in *C.elegans*: extrachromosomal maintenance and integration of transforming sequences. *EMBO J.* **10**, 3959-3970, (1991).

63 Swierczek, N. A., Giles, A. C., Rankin, C. H. & Kerr, R. A. High-throughput behavioral analysis in *C. elegans*. *Nat. Methods* **8**, 592-598, (2011).

64 Chronis, N., Zimmer, M. & Bargmann, C. I. Microfluidics for in *vivo* imaging of neuronal and behavioral activity in *Caenorhabditis elegans*. *Nat. Methods* **4**, 727-731, (2007).

65 Wang, W. *et al.* Off-response in ASH neurons evoked by $CuSO_4$ requires the TRP channel OSM-9 in *Caenorhabditis elegans*. *Biochem. Biophys. Res. Commun.* **461**, 463-468, (2015).

Supplementary Figures

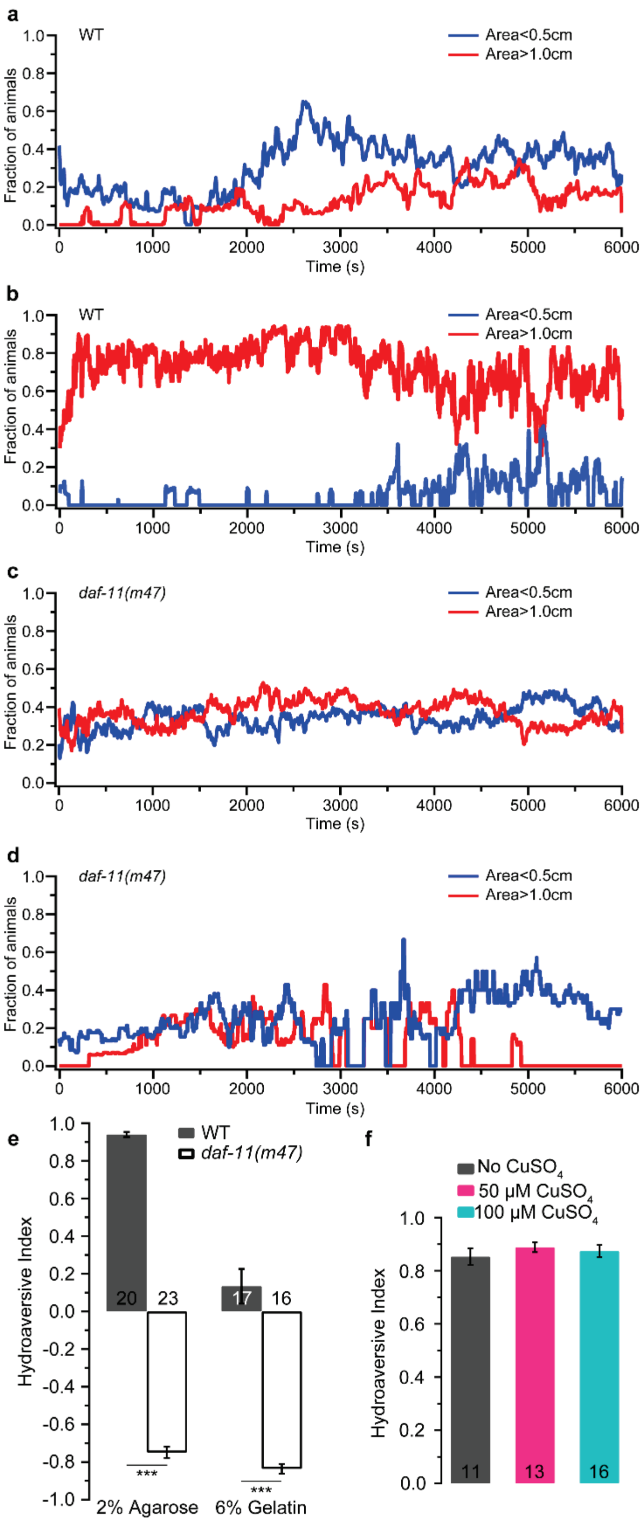

Supplementary Figure 1 | The wild-type N2 worms showed a preference for drier area in the WSA assay. a, b, Distribution of wild-type N2 worms on a regulative agar plate without water content gradient (WCG) (**a**) or a wedge-shaped agar plate with

WCG (**b**) from 0 s to 6000 s. **c, d,** Distribution of *daf-11(m47)* mutants on an agar plate without (**c**) or with (**d**) WCG from 0 s to 6000 s. (**e**) Hydroaversive indexes of wild-type worms and *daf-11(m47)* mutants in the WAS assay by use of 2% agarose and 6% gelatin respectively. (**f**) Hydroaversive index of wild-type worms in the WAS assay when 50 mM or 100 mM $CuSO_4$ was added. Statistics: *** $P \leq 0.001$ in comparison with each N2 control (*Student's t*-test or *Mann-Whitney Rank Sum*-test according to the normality of the data distribution). Error bars indicate the SEMs. The number of independent tests is shown for each test on each bar. WT, wild-type; H.A., hydroaversive.

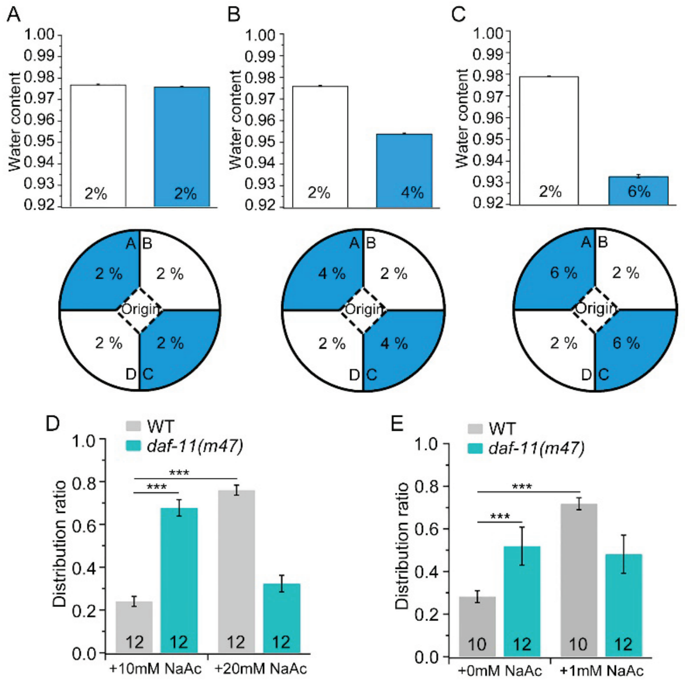

Supplementary Figure 2 | The four-quadrant agar assays. a - c Water content of 2% - 2%, 4% - 2% and 6% - 2% quadrant agar plates, respectively. Error bars indicate the SEMs. The lower panels show schematic diagrams. Figures are not drawn to scale. The number of independent tests is shown for each test on each bar. **d** and **e**, Distribution ratios of wild-type animals and *daf-11(m47)* mutants on the 2 % - 2 % agarose four-quadrant plates in which different concentrations of Na^+ were added into two opposite quadrants. The concentrations of Na^+ are 10 and 20 mM in **d**, and 0 and 1 mM in **e**. The number of independent tests is shown for each test on each bar. Statistics: *** $P \leq 0.001$

compared as indicated (*Student's t*-test or *Mann-Whitney Rank Sum*-test according to the normality of the data distribution). Error bars indicate the SEMs. The number of independent tests is shown for each test on each bar. WT, wild-type.

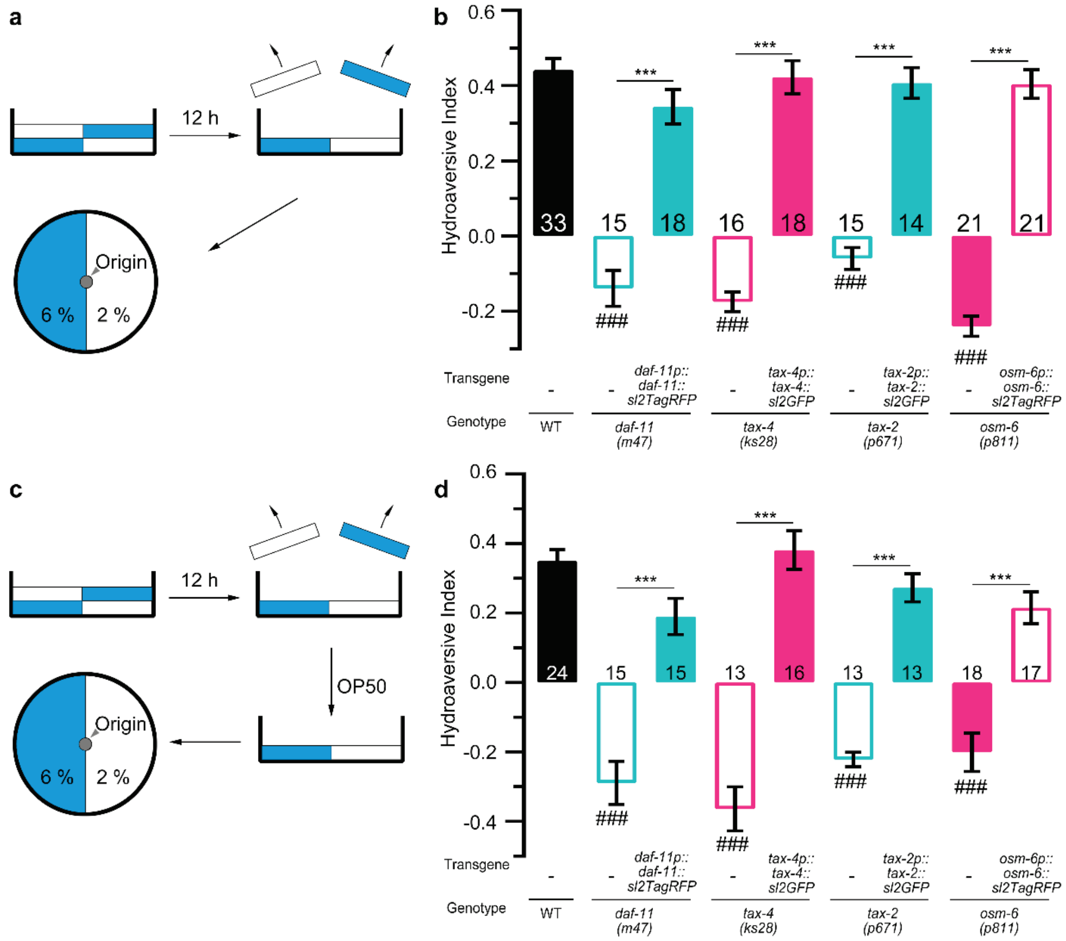

Supplementary Figure 3 | The assays on 6% - 2% agarose plates with long-term diffusion. a and c, Schema of the assays on the 6% - 2% agarose plates with long-term (> 12 hours) diffusion, in presence of (**a**) or in absence of (**c**) food. Two layers of 6% and 2% agarose was poured and diffused for over 12h. **b**, Hydroaversive indexes of *daf-11(m47)*, *tax-2(p671)*, *tax-4(ks28)*, *osm-6(p811)* and the genetically rescued worms in assay model **a** (with food). **d**, Hydroaversive indexes of *daf-11(m47)*, *tax-2(p671)*, *tax-4(ks28)*, *osm-6(p811)* and the genetically rescued worms in assay model c (without food).

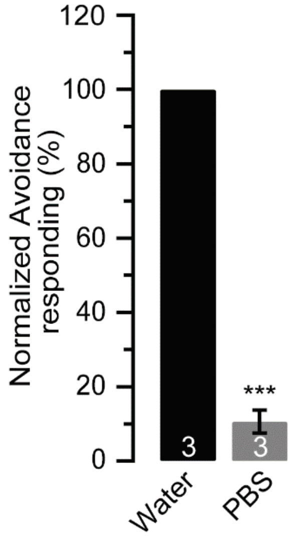

Supplementary Figure 4 | Normalized avoidance indexes of wild-type worms in drop test upon challenge of ultra-pure water and PBS. Statistics: *** $P \leq 0.001$ in comparison with N2 control (*Student's t*-test or *Mann-Whitney Rank Sum*-test according to the normality of the data distribution). Error bars indicate the SEMs. The number of independent tests is shown for each test on each bar.

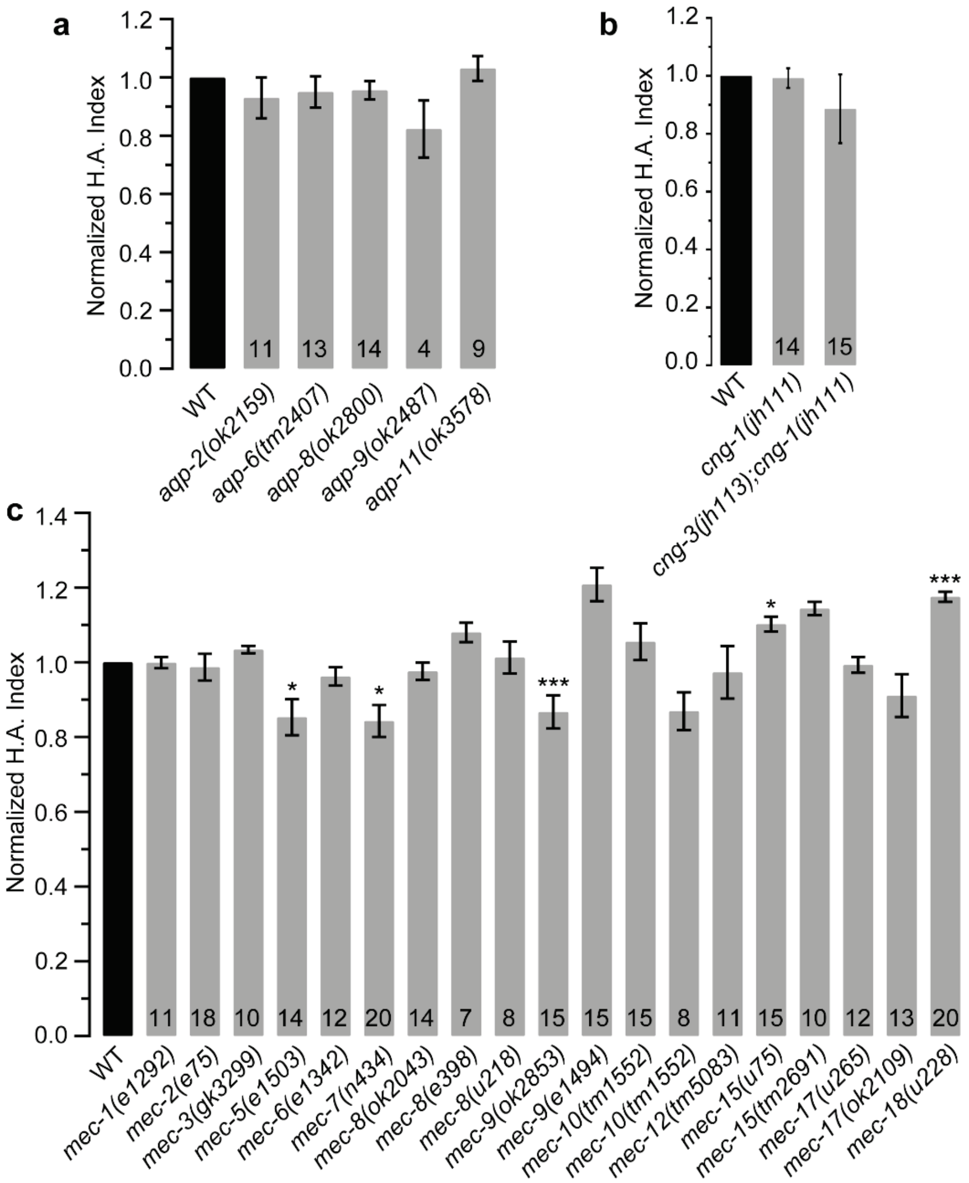

Supplementary Figure 5 | Hydropreference of mutants of ion and water channels. Worm hydropreference was tested by the wedge-shaped agar paradigm. **a-d** Normalized hydrotaxis indexes of mutants of aquaporins (**a**), cyclic nucleotide-gated (*cng*) channels (**b**), and mechanosensory abnormality (*mec)* channels (**c**), respectively. Data for all genotypes are normalized to their own wild type N2 control which is shown as only one bar. The number of independent tests is shown for each genotype on each bar. Statistics: * $P \leq 0.05$, ** $P \leq 0.01$ and *** $P \leq 0.001$ in comparison with each N2 control (*Student's t*-test or *Mann-Whitney Rank Sum*-test according to the normality of the data distribution). Error bars indicate the SEMs. WT, wild-type; H.A., hydroaversive.

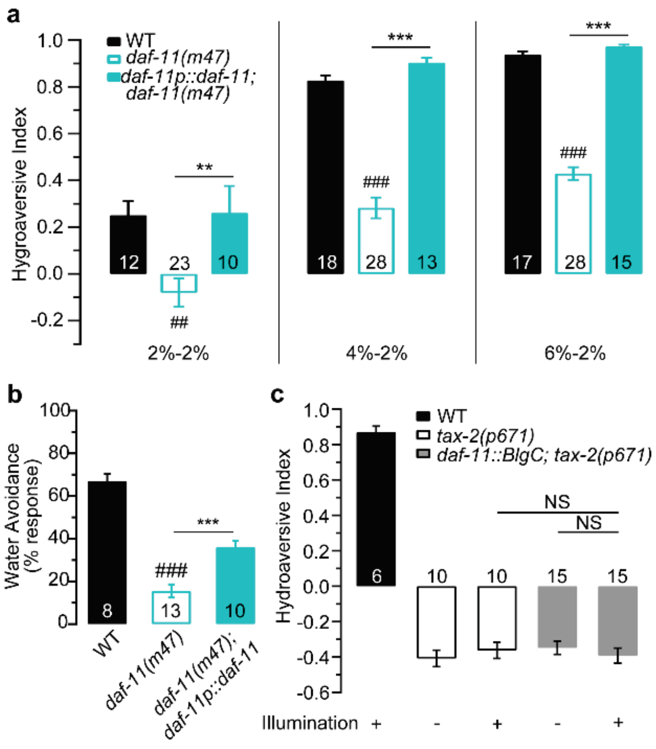

Supplementary Figure 6 | DAF-11 – TAX-2/TAX-4 signalling pathway involves in hydrosesation. a, b, *daf-11* mutants exhibited hydroaversive defects examined by the quadrant agar test and the drop test. Hydroaversive indexes of *daf-11(m47)* and the genetic rescued worms in the quadrant agar test paradigm (**a**) and ratios of water avoidance in *daf-11(m47)* mutants and the genetic rescued worms assayed by the drop test (**b**). Extrachromosomal expression of *daf-11* genomic DNA is driven by its own promoter. **c,** Photo-activation of BlgC in *daf-11*-expressing neurons did not rescue hydrotaxis defect in *tax-2(p671)* mutants in the WSA assay. Statistics: $^{###}$ $P \leq 0.001$ compared with each N2 control, *** $P \leq 0.001$ compared as indicated, NS, not significant (*Student's t*-test or *Mann-Whitney Rank Sum* –test according to the normality of the data distribution). Error bars indicates the SEMs. The number of independent tests is indicated for each test. WT, wild-type; H.A., hydroaversive.

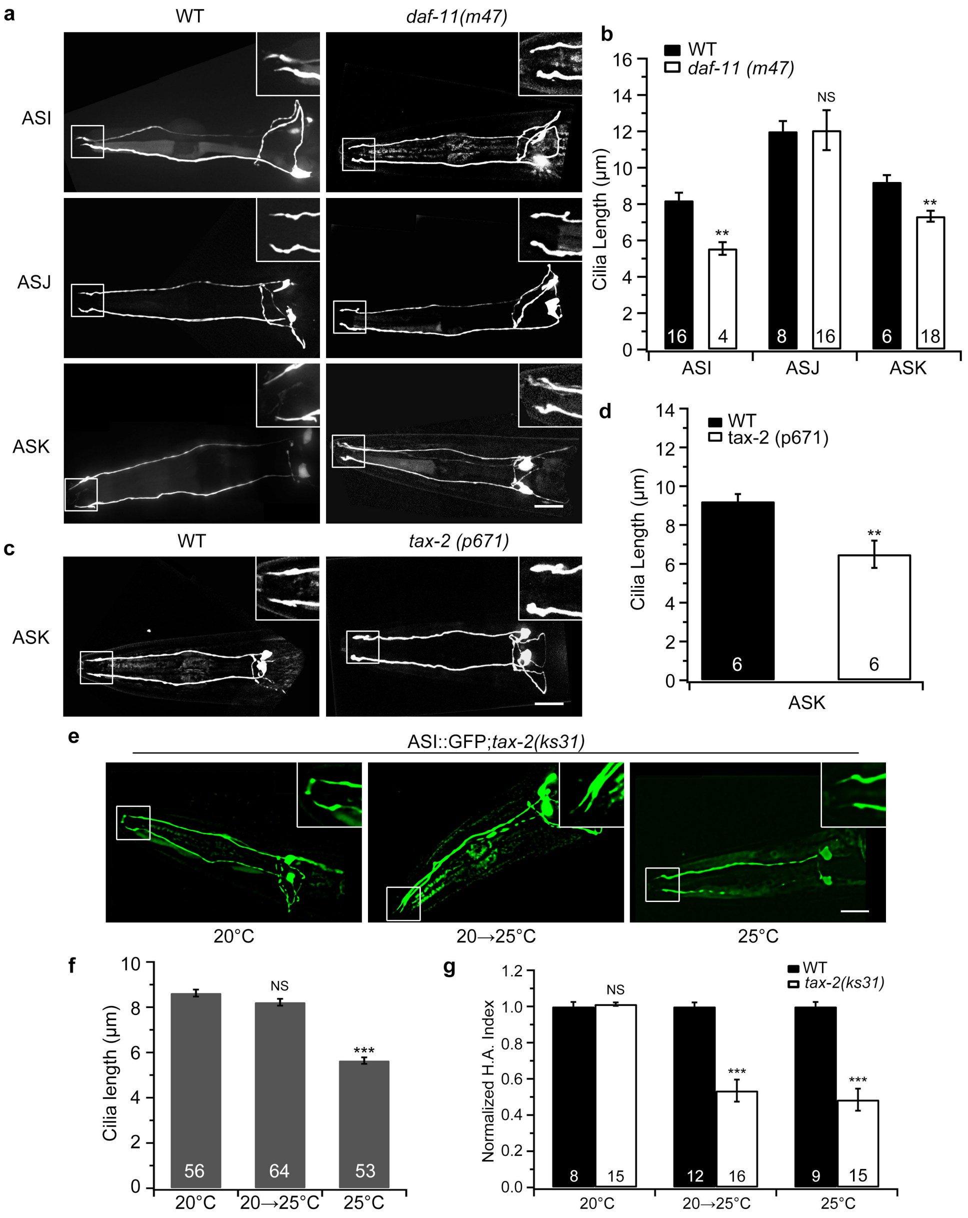

Supplementary Figure 7 | DAF-11 – TAX-2/TAX-4 signalling regulates cilium development in ciliated sensory neurons. a, Confocal images of cilium structures of ASI, ASK and ASJ neurons in wild-type and *daf-11(m47)* worms. **b,** Summation of the cilium length of ASI, ASK and ASJ neurons in wild-type N2 and *daf-11(m47)* worms. **c,** Confocal images of cilium structures of ASK neurons in wild-type and *tax-2(p671)* worms. **d,** The cilium length of ASK neurons in N2 worms and *tax-2(p671)* mutants. **e,** Confocal images of cilium structures of ASI neurons in *tax-2(ks31)* mutants treated with adult temperature shift. Animals were allowed to grow at permissive temperature (20 °C) till young adult stage then shifted to restrictive temperature (25 °C) for 24h. **f,** Summation of the cilium length of worms grown under different growing conditions.

g, Normalized hydroaversive indexes of temperature sensitive strain *tax-2(ks31)* raised in different temperature conditions tested by the wedge-shaped agar paradigm. 20 °C and 25 °C, worms raised at normal permissive temperature 20 °C and restrictive temperature 25 °C respectively. 20 → 25 °C, worms treated with young adult temperature shift (from 20 °C to 25 °C at young adult) for 12 hours. Statistics: ** $P \leq 0.01$, *** $P \leq 0.001$ in comparison with each wild-type control (*Student's t*-test or *Mann-Whitney Rank Sum*-test according to the normality of the data distribution). NS, not significant. Error bars indicate the SEMs. The number of measured neurons or independent test for each genotype is indicated. WT, wild-type; H.A., hydroaversive.

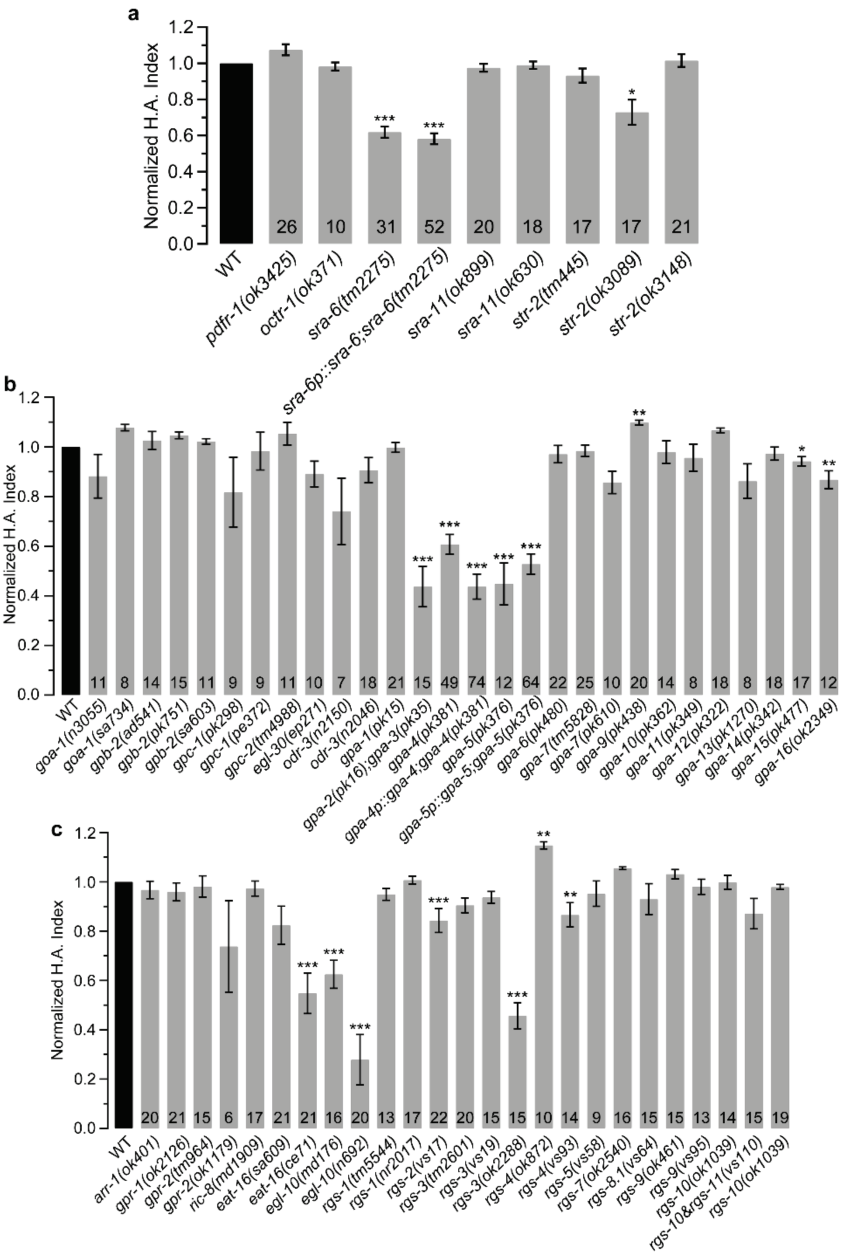

Supplementary Figure 8 | Normalized hydropreference of mutants of G-protein-coupled receptors, G proteins and G protein regulatory proteins. Worm hydropreference was tested by the wedge-shaped agar paradigm. **a–c** Normalized hydrotaxis indexes of mutants of G-protein-coupled receptors (**a**), G proteins (**b**) and G protein regulatory proteins (**c**). Data of all genotypes are normalized to their own wild-type N2 control that is shown as only one bar. The number of independent tests is indicated for each genotype on each bar. Statistics: * $P \leq 0.05$, ** $P \leq 0.01$, *** $P \leq 0.001$ compared with each N2 control (*Student's t*-test or *Mann-Whitney Rank Sum* –test depending on the normality of the data distribution). Error bars indicate the SEMs. WT, wild-type; H.A., hydroaversive.

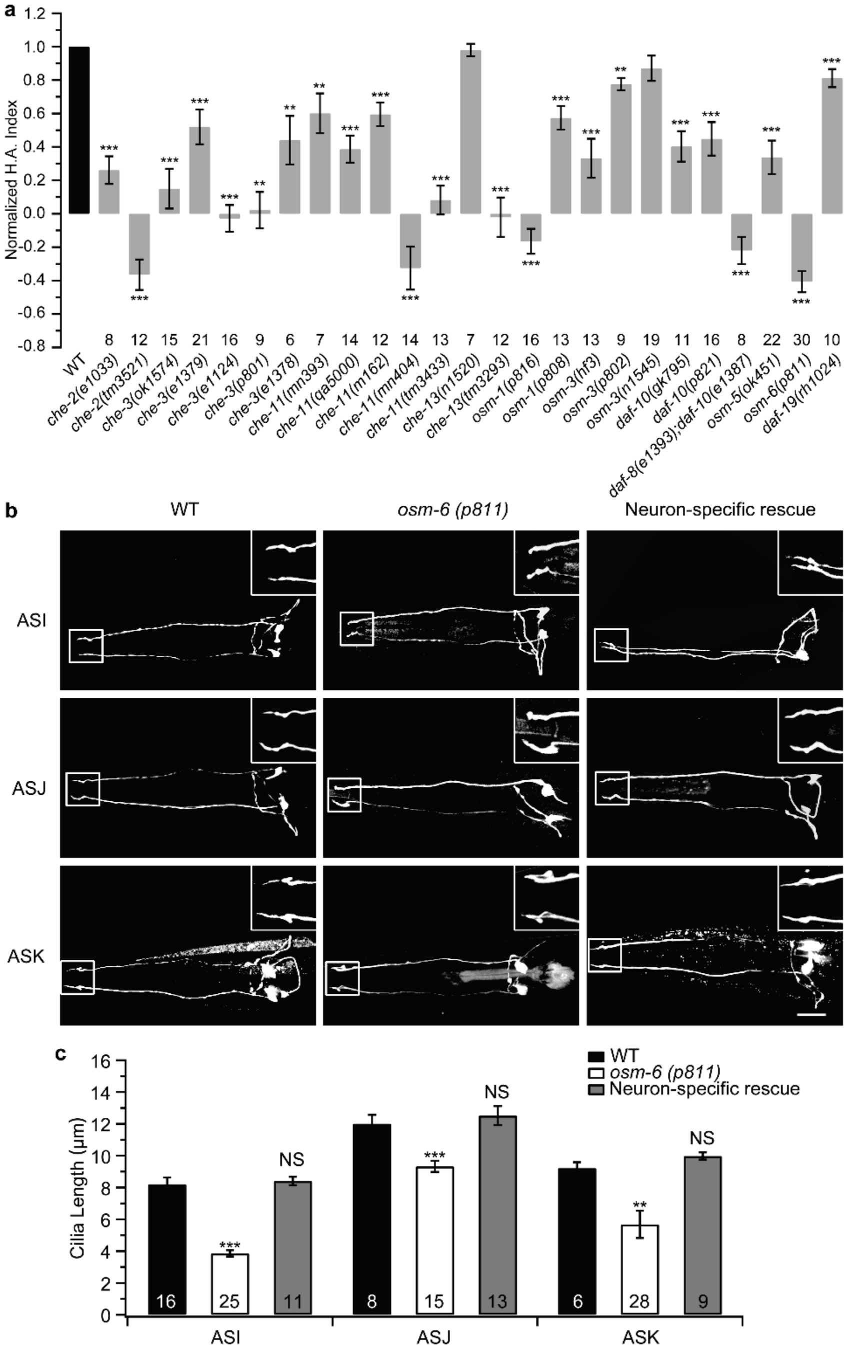

Supplementary Figure 9 | Ciliated sensory neurons involve in hydrosensation. a, Hydrotaxis of mutants bearing with defect in sensory cilium development. The worm hydropreference was assayed by the wedge-shaped agar paradigm. H.A. indexes of mutants are normalized to each wild-type N2 control shown as only one bar. The number of independent tests is indicated for each genotype under each bar. **b,** Confocal images of cilium structures of ASI, ASK and ASJ neurons in wild-type animals, *osm-6(p811)* mutants and genetically rescued worms by ectoexpression of *osm-6* genomic

DNA driven by *gpa-4* (ASI), *srh-11* (ASJ) and *sra-9* (ASK) promoters, respectively. **c,** Summation of the cilium length of ASI, ASK and ASJ neurons in each genotype. The number of measured neurons is indicated on each bar. Statistics: ** $P \leq 0.01$, *** $P \leq 0.001$ and NS, not significant in comparison with each wild-type N2 control (*Student's t*-test or *Mann-Whitney Rank Sum*-test according to the normality of the data distribution). Error bars indicate the SEMs. WT, wild-type; H.A., hydroaversive.

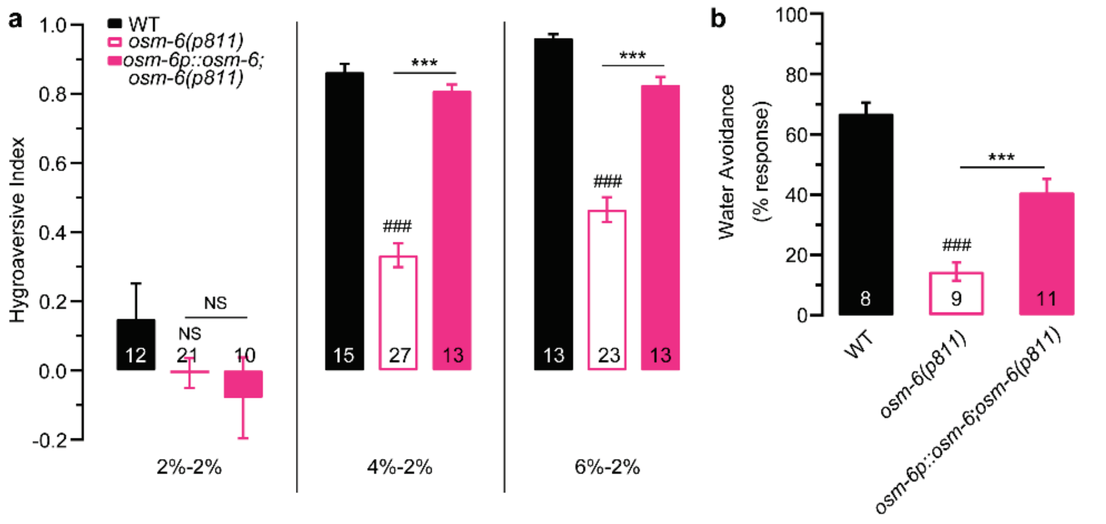

Supplementary Figure 10 | *osm-6* mutants exhibit hydroaversive defect examined by quadrant and drop tests. Extrachromosomal expression of *osm-6* genomic DNA was driven by its own promoter. **a,** Hydroaversive indexes of *osm-6 (p811)* and the genetically rescued worms in the four-quadrant agar test paradigm. **b,** Ratios of water avoidance in *osm-6 (p811)* mutants and the rescued worms in the drop test. Statistics: $^{###}$ $P \leq 0.001$ compared with each wild-type N2 control, *** $P \leq 0.001$ compared as indicated (*Student's t*-test or *Mann-Whitney Rank Sum* test according to the normality of the data distribution). Error bars indicate the SEMs. The number of independent tests is indicated for each genotype on each bar. WT, wild-type.

Supplementary Video Legends

Supplementary Movie 1 | A dry drop test of a wild-type animal upon stimulation by a drop of ultrapure water. An adult wild-type N2 worm exhibited rhythmic sinusoidal backward locomotion when its nose touched a local spot instilled with a micro drop (a few hundred nanolitres) of ultrapure water that had absorbed into agar. Yellow circle encloses the area instilled with water drop.

Supplementary Movie 2 | A drop test of wild-type animal upon stimulation by a drop of ultrapure water. An adult wild-type N2 worm displayed rhythmic sinusoidal backward locomotion when its nose touched a micro drop (a few hundred nanolitres) of ultra-pure water.

Supplementary Movie 3 | A dry drop test of wild-type animal upon challenge by a drop of PBS. An adult wild-type N2 worm exhibited no response when its nose touched a local spot instilled with a micro drop (a few hundred nanolitres) of phosphate buffered saline (PBS) that had absorbed into agar. Yellow circle encloses the area instilled with solution drop.

Supplementary Movie 4 | A drop test of wild-type animal upon challenge by a drop of PBS. An adult wild-type N2 worm displayed no response when its nose touched a micro drop (a few hundred nanolitres) of phosphate buffered saline (PBS).

Supplementary Table

Supplymentary Table 1 | Mutant and transgenic worms used in the study.

Strain	Genotype
DR47	*daf-11(m47)* V
PR671	*tax-2(p671)* I
FK103	*tax-4(ks28)* III
FK104	*tax-2(ks31)* I
FG7	*grk-2(gk268)* III
tm2483	*str-3(tm2483)* IV
KG524	*gsa-1(ce94)* I
NL334	*gpa-2(pk16)* V
NL335	*gpa-3(pk35)* V
PR811	*osm-6(p811)* V
RB1715	*aqp-2(ok2159)* II
tm2407	*aqp-6(tm2407)* V
RB2115	*aqp-8(ok2800)* X
RB1914	*aqp-9(ok2487)* I
RB2570	*aqp-11(ok3578)* III
tm717	*del-4(tm717)* I
RB1156	*C46A5.2(ok1187)* IV
RB1818	*C18B2.6(ok2353)* X
CB1292	*mec-1(e1292)* V
CB75	*mec-2(e75)* X
VC3249	*mec-3(gk3299)* IV
CB1503	*mec-5(e1503)* X
CB1472	*mec-6(e1342)* I
TU300	*mec-7(n434)* X
VC1594	*mec-8(ok2043)* I
CB398	*mec-8(e398)* I
SP1560	*mec-8(u218)* I
RB2140	*mec-9(ok2853)* V
CB1494	*mec-9(e1494)* V
RB1115	*mec-10(ok1104)* X
ZB2551	*mec-10(tm1552)* X
tm5083	*mec-12(tm5083)* III
TU75	*mec-15(u75)* II
tm2691	*mec-15(tm2691)* II
TU265	*mec-17(u265)* IV
RB1696	*mec-17(ok2109)* IV

TU228	*mec-18(u228)* X
RB1052	*trpa-1(ok999)* IV
VC2435	*trpa-2(ok3189)* I
TQ225	*trp-1(sy690)* III
VC160	*trp-1(ok323)* III
TQ296	*trp-4(sy695)* I
VC818	*trp-4(gk341)* I
CX4533	*ocr-1(ok132)* V
CX4534	*ocr-1(ak46)* V
LX980	*ocr-4(vs137)* IV; *ocr-1(ok132)* V
LX982	*ocr-4(vs137)*; *ocr-2(ak47)* IV; *ocr-1(ok132)* V
LX981	*ocr-4(vs137)*; *ocr-2(ak47)* IV
LX845	*ocr-2(ak47)* IV; *ocr-1(ok132)* V
RB1374	*ocr-3(ok1559)* X
LX950	*ocr-4(VS 137)* IV
RB753	*lov-1(ok522)* II
CX10	*osm-9(ky10)* IV
tm5418	*osm-9(tm5418)* IV
tm5536	*osm-9(tm5536)* IV
EJ26	*gon-2(q362)* I
CZ9957	*gtl-2(n2618)* IV
KJ461	*cng-1(jh111)* V
KJ5560	*cng-3(jh113)* IV; *cng-1(jh111)* V
MT8626	*goa-1(n3055)* I
DG1856	*goa-1(sa734)* I
DA541	*gpb-2(ad541)* I
NL2001	*gpb-2(pk751)* I
JT603	*gpb-2(sa603)* I
NL792	*gpc-1(pk298)* X
JN372	*gpc-1(pe372)* X
tm4988	*gpc-2(tm4988)* I
CE1047	*egl-30(ep271)* I
CX2205	*odr-3(n2150)* V
MT4810	*odr-3(n2046)* V
NL332	*gpa-1(pk15)* V
NL348	*gpa-2(pk16)*; *gpa-3(pk35)* V
NL790	*gpa-4(pk381)* IV
ZXW891	*gpa-4(pk381)* IV; *hkdEx891* [*gpa-4p::gpa-4::sl2e::TagRFP-T; vha-6::gfp*]
ZXW892	*gpa-4(pk381)* IV; *hkdEx892* [*gpa-4p::gpa-4::sl2e::TagRFP-T; vha-6::gfp*]
ZXW893	*gpa-4(pk381)* IV; *hkdEx893* [*gpa-4p::gpa-4::sl2e::TagRFP-T; vha-6::gfp*]
ZXW894	*gpa-4(pk381)* IV; *hkdEx894* [*gpa-4p::gpa-4::sl2e::TagRFP-T; vha-6::gfp*]

NL1137	*gpa-5(pk376)* X
ZXW945	*gpa-5(pk376)* X; *hkdEx945* [*gpa-5p::gpa-5::sl2e-TagRFP-t*; *vha-6::gfp*]
ZXW946	*gpa-5(pk376)* X; *hkdEx946* [*gpa-5p::gpa-5::sl2e-TagRFP-t*; *vha-6::gfp*]
ZXW947	*gpa-5(pk376)* X; *hkdEx947* [*gpa-5p::gpa-5::sl2e-TagRFP-t*; *vha-6::gfp*]
NL1146	*gpa-6(pk480)* X
tm5828	*gpa-7(tm5828)* IV
NL795	*gpa-7(pk610)* IV
NL793	*gpa-9(pk438)* V
NL1147	*gpa-10(pk362)* V
NL787	*gpa-11(pk349)* II
NL594	*gpa-12(pk322)* X
NL2330	*gpa-13(pk1270)* V
NL788	*gpa-14(pk342)* I
NL797	*gpa-15(pk477)* I
RB1816	*gpa-16(ok2349)* I
RB660	*arr-1(ok401)* X
VC1670	*gpr-1(ok2126)* I
tm964	*gpr-2(tm964)* III
RB1150	*gpr-2(ok1179)* III
RM2209	*ric-8(md1909)* IV
JT609	*eat-16(sa609)* I
KG571	*eat-16(ce71)* I
MT8504	*egl-10(md176)* V
MT1443	*egl-10(n692)* V
tm5544	*rgs-1(tm5544)* III
LX147	*rgs-1(nr2017)* III
LX160	*rgs-2(vs17)* X
tm2601	*rgs-3(tm2601)* II
LX242	*rgs-3(vs19)* II
RB1780	*rgs-3(ok2288)* II
RB968	*rgs-4(ok872)* II
LX604	*rgs-4(vs93)* II
LX475	*rgs-5(vs58)* III
RB1937	*rgs-7(ok2540)* X
LX543	*rgs-8.1(vs64)* X
RB699	*rgs-9(ok461)* X
LX606	*rgs-9(vs95)* X
RB1077	*rgs-10(ok1039)* X
LX733	*rgs-10&rgs-11(vs110)* X
VC700	*rgs-10(ok1039)* X
CB1033	*che-2(e1033)* X

tm3521	*che-2(tm3521)* X
VC1080	*che-3(ok1574)* I
CB1379	*che-3(e1379)* I
CB1124	*che-3(e1124)* I
PR801	*che-3(p801)* I
CB1378	*che-3(e1378)* I
SP1409	*che-11(mn393)* V
XA5000	*che-11(qa5000)* V
DR512	*che-11(m162)* V
SP1416	*che-11(mn404)* V
tm3433	*che-11(tm3433)* V
MT3575	*che-13(n1520)* I
tm3293	*che-13(tm3293)* I
PR816	*osm-1(p816)* X
PR808	*osm-1(p808)* X
PH27	*osm-3(hf3)* IV
PR802	*osm-3(p802)* IV
MT3631	*osm-3(n1545)* IV
VC1641	*daf-10(gk795)* IV
PR821	*daf-10(p821)* IV
DR184	*daf-8(e1393)* I; *daf-10(e1387)* IV
VC265	*osm-5(ok451)* X
PR811	*osm-6(p811)* V
OE3059	*daf-19(rh1024)* II
ZXW640	*daf-11(m47)* V; *hkdEx640* [*daf-11p::daf-11::sl2e::TagRFP-t; vha-6p::GFP*]
ZXW860	*tax-2(p671)* I; *hkdEx860* [*tax-2p::tax-2::sl2e:::GFP; vha-6p::gfp*]
ZXW861	*tax-2(p671)* I; *hkdEx861* [*tax-2p::tax-2::sl2e:::GFP; vha-6p::gfp*]
ZXW862	*tax-2(p671)* I; *hkdEx862* [*tax-2p::tax-2::sl2e::GFP; vha-6p::gfp*]
ZXW863	*tax-4(ks28)* III; *hkdEx863* [*tax-4p::tax-4::sl2e::GFP; myo-3p::mKate2*]
ZXW866	*daf-11(m47)* V; *tax-2(p671)* I; *hkdEx866* [*daf-11p::daf-11::sl2TagRFP-t; tax-2p::tax-2::sl2gfp; vha-6p::gfp*]
ZXW870	*daf-11(m47)* V; *tax-2(p671)* I; *hkdEx870* [*daf-11p::daf-11::sl2TagRFP-t; vha-6p::gfp*]
ZXW871	*daf-11(m47)* V; *tax-2(p671)* I; *hkdEx871* [*daf-11p::daf-11::sl2TagRFP-t; vha-6p::gfp*]
ZXW872	*daf-11(m47)* V; *tax-2(p671)* I; *hkdEx872* [*tax-2p::tax-2::sl2e::gfp; vha-6p::gfp*]
ZXW873	*daf-11(m47)* V; *tax-2(p671)* I; *hkdEx873* [*tax-2p::tax-2e::sl2gfp; vha-6p::gfp*]
ZXW874	*daf-11(m47)* V; *tax-2(p671)* I; *hkdEx874* [*tax-2p::tax-2e::sl2gfp; vha-6p::gfp*]
ZXW875	*grk-2(gk268)* III; *hkdEx875* [*grk-2p::grk-2::sl2e::TagRFP-t; vha-6p::gfp*]
ZXW876	*grk-2(gk268)* III; *hkdEx876* [*grk-2p::grk-2::sl2e::TagRFP-t; vha-6p::gfp*]
ZXW877	*grk-2(gk268)* III; *hkdEx877* [*grk-2p::grk-2::sl2e::TagRFP-t; vha-6p::gfp*]
ZXW878	*grk-2(gk268)* III*; hkdEx878* [*grk-2p::grk-2::sl2e::TagRFP-t; vha-6p::gfp*]

ZXW879	*str-3(tm2483)* IV; *hkdEx879* [*str-3p::str-3::sl2e::TagRFP-t; vha-6p::gfp*]
ZXW880	*str-3(tm2483)* IV; *hkdEx880* [*str-3p::str-3::sl2e::TagRFP-t; vha-6p::gfp*]
ZXW881	*str-3(tm2483)* IV; *hkdEx881* [*str-3p::str-3::sl2e::TagRFP-t; vha-6p::gfp*]
ZXW882	*gsa-1(ce94)* I; *hkdEx882* [*gsa-1p::gsa-1::sl2e::TagRFP-t; vha-6p::gfp*]
ZXW883	*gsa-1(ce94)* I; *hkdEx883* [*gsa-1p::gsa-1::sl2e::TagRFP-t; vha-6p::gfp*]
ZXW884	*gsa-1(ce94)* I; *hkdEx884* [*gsa-1p::gsa-1::sl2e::TagRFP-t; vha-6p::gfp*]
ZXW885	*gpa-2(pk16)* V; *hkdEx885* [*gpa-2p::gpa-2::sl2e::TagRFP-t; vha-6p::gfp*]
ZXW886	*gpa-2(pk16)* V; *hkdEx886* [*gpa-2p::gpa-2::sl2e::TagRFP-t; vha-6p::gfp*]
ZXW887	*gpa-2(pk16)* V; *hkdEx887* [*gpa-2p::gpa-2::sl2e::TagRFP-t; vha-6p::gfp*]
ZXW888	*gpa-3(pk35)* V; *hkdEx888* [*gpa-3p::gpa-3::sl2e::TagRFP-T; vha-6p::gfp*]
ZXW889	*gpa-3(pk35)* V; *hkdEx889* [*gpa-3p::gpa-3::sl2e::TagRFP-T; vha-6p::gfp*]
ZXW890	*gpa-3(pk35)* V; *hkdEx890* [*gpa-3p::gpa-3::sl2e::TagRFP-T; vha-6p::gfp*]
ZXW891	*gpa-4(pk381)* IV; *hkdEx891* [*gpa-4p::gpa-4::sl2e::TagRFP-T; vha-6p::gfp*]
ZXW892	*gpa-4(pk381)* IV; *hkdEx892* [*gpa-4p::gpa-4::sl2e::TagRFP-T; vha-6p::gfp*]
ZXW893	*gpa-4(pk381)* IV; *hkdEx893* [*gpa-4p::gpa-4::sl2e::TagRFP-T; vha-6p::gfp*]
ZXW894	*gpa-4(pk381)* IV; *hkdEx894* [*gpa-4p::gpa-4::sl2e::TagRFP-T; vha-6p::gfp*]
ZXW895	*daf-11(m47)* V; *hkdEx895* [*gpa-4p::daf-11::sl2e::TagRFP-t; vha-6p::gfp*]
ZXW896	*daf-11(m47)* V; *hkdEx896* [*gpa-4p::daf-11::sl2e::TagRFP-t; vha-6p::gfp*]
ZXW897	*daf-11(m47)* V; *hkdEx897* [*gpa-4p::daf-11::sl2e::TagRFP-t; vha-6p::gfp*]
ZXW898	*daf-11(m47)* V; *hkdEx898* [*sra-9p::daf-11::sl2e::TagRFP-t; vha-6p::gfp*]
ZXW899	*daf-11(m47)* V; *hkdEx899* [*sra-9p::daf-11::sl2e::TagRFP-t; vha-6p::gfp*]
ZXW900	*daf-11(m47)* V; *hkdEx900* [*sra-9p::daf-11::sl2e::TagRFP-t; vha-6p::gfp*]
ZXW901	*daf-11(m47)* V; *hkdEx901* [*srh-11p::daf-11::sl2e::TagRFP-t; vha-6p::gfp*]
ZXW902	*daf-11(m47)* V; *hkdEx903* [*srh-11p::daf-11::sl2e::TagRFP-t; vha-6p::gfp*]
ZXW903	*daf-11(m47)* V; *hkdEx903* [*srh-11p::daf-11::sl2e::TagRFP-t; vha-6p::gfp*]
ZXW904	*osm-6(p811)* V; *hkdEx904* [*osm-6p::osm-6::SL2e::TagRFP-t; lin-44p::gfp*]
ZXW905	*osm-6(p811)* V; *hkdEx905* [*osm-6p::osm-6::SL2e::TagRFP-t; lin-44p::gfp*]
ZXW906	*osm-6(p811)* V; *hkdEx906* [*osm-6p::osm-6::SL2e::TagRFP-t; lin-44p::gfp*]
ZXW907	*osm-6(p811)* V; *hkdEx907* [*tax-2p::osm-6::sl2e-TagRFP-t; lin-44p::gfp*]
ZXW908	*osm-6(p811)* V; *hkdEx908* [*tax-2p::osm-6::sl2e-TagRFP-t; lin-44p::gfp*]
ZXW909	*osm-6(p811*) V; *hkdEx909* [*gpa-4p::osm-6::sl2e-TagRFP-t; lin-44p::gfp*]
ZXW910	*osm-6(p811)* V; *hkdEx910* [*srh-11p::osm-6::sl2e-TagRFP-t; lin-44p::gfp*]
ZXW911	*osm-6(p811*) V; *hkdEx911* [*srh-11p::osm-6::sl2e-TagRFP-t; lin-44p::gfp*]
ZXW912	*osm-6(p811)* V; *hkdEx912* [*srh-11p::osm-6::sl2e-TagRFP-t; lin-44p::gfp*]
ZXW913	*osm-6(p811)* V; *hkdEx913* [*sra-9p::osm-6::sl2e-TagRFP-t; lin-44p::gfp*]
ZXW914	*osm-6(p811)* V; *hkdEx914* [*sra-9p::osm-6::sl2e-TagRFP-t; lin-44p::gfp*]
ZXW915	*osm-6(p811)* V; *hkdEx915* [*sra-9p::osm-6::sl2e-TagRFP-t; lin-44p::gfp*]
ZXW916	*osm-6(p811)* V; *hkdEx916* [*sra-9p::osm-6::sl2e-TagRFP-t; lin-44p:gfp*]
ZXW917	*osm-6(p811)* V; *hkdEx917* [*odr-10p::osm-6::sl2e-TagRFP-t; lin-44p:gfp*]
ZXW918	*osm-6(p811*) V; *hkdEx918* [*odr-10p::osm-6::sl2e-TagRFP-t; lin-44p:gfp*]
ZXW919	*osm-6(p811)* V; *hkdEx919* [*str-1p::osm-6::sl2e-TagRFP-t; lin-44p::gfp*]

ZXW920	*osm-6(p811)* V; *hkdEx920* [*str-1p::osm-6::sl2e-TagRFP-t; lin-44p::gfp*]
ZXW921	*osm-6(p811)* V; *hkdEx921* [*str-1p::osm-6::sl2e-TagRFP-t; lin-44p::gfp*]
ZXW922	*osm-6(p811)* V; *hkdEx922* [*gcy-8p::osm-6::sl2e-TagRFP-t; lin-44p::gfp*]
ZXW923	*osm-6(p811)* V; *hkdEx923* [*gcy-8p::osm-6::sl2e-TagRFP-t; lin-44p::gfp*]
ZXW924	*osm-6(p811)* V; *hkdEx924* [*gcy-8p::osm-6::sl2e-TagRFP-t; lin-44p::gfp*]
ZXW925	*osm-6(p811)* V; *hkdEx925* [*odr-3p::osm-6::sl2e-TagRFP-t; lin-44p::gfp*]
ZXW926	*osm-6(p811)* V; *hkdEx926* [*odr-3p::osm-6::sl2e-TagRFP-t; lin-44p::gfp*]
ZXW927	*osm-6(p811)* V; *hkdEx927* [*odr-3p::osm-6::sl2e-TagRFP-t; lin-44p::gfp*]
ZXW928	*osm-6(p811)* V; *hkdEx928* [*odr-3p::osm-6::sl2e-TagRFP-t; lin-44p::gfp*]
ZXW929	*osm-6(p811)* V; *hkdEx929* [*ceh-36p::osm-6::sl2e-TagRFP-t; lin-44p::gfp*]
ZXW930	*osm-6(p811)* V; *hkdEx930* [*ceh-36p::osm-6::sl2e-TagRFP-t; lin-44p::gfp*]
ZXW931	*osm-6(p811)* V; *hkdEx931* [*ceh-36p::osm-6::sl2e-TagRFP-t; lin-44p::gfp*]
ZXW932	*osm-6(p811)* V; *hkdEx932* [*gcy-33p::osm-6::sl2e-TagRFP-t; vha-6p::gfp*]
ZXW933	*osm-6(p811)* V; *hkdEx933* [*gcy-33p::osm-6::sl2e-TagRFP-t; vha-6p::gfp*]
ZXW934	*osm-6(p811)* V; *hkdEx934* [*gcy-33p::osm-6::sl2e-TagRFP-t; vha-6p::gfp*]
ZXW935	*osm-6(p811)* V; *hkdEx935* [*ops-1p::osm-6::sl2e-TagRFP-t; vha-6p::gfp*]
ZXW936	*osm-6(p811)* V; *hkdEx936* [*ops-1p::osm-6::sl2e-TagRFP-t; vha-6p::gfp*]
ZXW937	*osm-6(p811)* V; *hkdEx937* [*ops-1p::osm-6::sl2e-TagRFP-t; vha-6p::gfp*]
ZXW938	*daf-11(m47)* V; *hkdEx938* [*daf-11p::Blgc::GFP; lin-44p::gfp*]
ZXW939	*daf-11(m47)* V; *hkdEx939* [*gpa-4p::Blgc::GFP; myo-3p::mkate2*]
ZXW940	*daf-11(m47)* V; *hkdEx940* [*gpa-4p::Blgc::GFP; myo-3p::mkate2*]
ZXW941	*daf-11(m47)* V; *hkdEx941* [*gpa-4p::Blgc::GFP; myo-3p::mkate2*]
ZXW942	*daf-11(m47)* V; *hkdEx942* [*srh-11p::Blgc::GFP; myo-3p::mkate2*]
ZXW943	*daf-11(m47)* V; *hkdEx943* [*sra-9p::Blgc::GFP; lin-44p::gfp*]
ZXW944	*daf-11(m47)* V; *hkdEx944* [*sra-9p::Blgc::GFP; lin-44p::gfp*]
ZXW945	*gpa-5(pk376)* X; *hkdEx945* [*gpa-5p::gpa-5::sl2e-TagRFP-t; vha-6p::gfp*]
ZXW946	*gpa-5(pk376)* X; *hkdEx946* [*gpa-5p::gpa-5::sl2e-TagRFP-t; vha-6p::gfp*]
ZXW947	*gpa-5(pk376)* X; *hkdEx947* [*gpa-5p::gpa-5::sl2e-TagRFP-t; vha-6p::gfp*]
ZXW948	*sra-6(tm2275)* II; *hkdEx948* [*sra-6p::sra-6 cDNA::sl2e-TagRFP-t; vha-6p::gfp*]
ZXW949	*sra-6(tm2275)* II; *hkdEx949* [*sra-6p::sra-6 cDNA::sl2e-TagRFP-t; vha-6p::gfp*]
ZXW950	*sra-6(tm2275)* II; *hkdEx950* [*sra-6p::sra-6 cDNA::sl2e-TagRFP-t; vha-6p::gfp*]
ZXW951	*hkdEx951* [*gpa-9p::gfp; vha-6p::gfp*]
ZXW952	*daf-11(m47)* V; *hkdEx952* [*gpa-9p::gfp; vha-6p::gfp*]
ZXW953	*osm-6(p811)* V; *hkdEx953* [*gpa-9p::gfp; vha-6p::gfp*]
ZXW954	*hkdEx954* [*gpa-4p::gfp; vha-6p::gfp*]
ZXW955	*daf-11(m47)* V; *hkdEx955* [*gpa-4p::gfp; vha-6p::gfp*]
ZXW956	*osm-6(p811)* V; *hkdEx956* [*gpa-4p::gfp; vha-6p::gfp*]
ZXW957	*hkdEx957* [*sra-9p::gfp; vha-6p::gfp*]
ZXW958	*daf-11(m47)* V; *hkdEx958* [*sra-9p::gfp; vha-6p::gfp*]
ZXW959	*osm-6(p811)* V; *hkdEx959* [*sra-9p::gfp; vha-6p::gfp*]
ZXW960	*hkdEx960* [*srd-1p::R-GECO1.0; lin-44p::gfp*]

ZXW961	*daf-11(m47)* V; *hkdEx961* [*gpa-4p::R-GECO1.0; lin-44p::gfp*]
ZXW962	*daf-11(m47)* V; *hkdEx962* [*daf-11p::daf-11::sl2e::TagRFP-t; gpa-4p::G-GECO1.1; lin-44p::gfp*]
ZXW963	*tax-4(ks28)* III; *hkdEx963* [*gpa-4p::tax-4 cDNA::sl2e::TagRFP-t; vha-6p::gfp*]
ZXW964	*tax-4(ks28)* III; *hkdEx964* [*sra-9p::tax-4 cDNA::sl2e::TagRFP-t; vha-6p::gfp*]
ZXW965	*tax-4(ks28)* III; *hkdEx965* [*sra-9p::tax-4 cDNA::sl2e::TagRFP-t; vha-6p::gfp*]
ZXW966	*tax-4(ks28)* III; *hkdEx966* [*sra-9p::tax-4 cDNA::sl2e::TagRFP-t; vha-6p::gfp*]
ZXW967	*tax-4(ks28)* III; *hkdEx967* [*srh-11p::tax-4 cDNA::sl2e::TagRFP-t; vha-6p::gfp*]
ZXW968	*tax-4(ks28)* III; *hkdEx968* [*srh-11p::tax-4 cDNA::sl2e::TagRFP-t; vha-6p::gfp*]
ZXW969	*tax-4(ks28)* III; *hkdEx969* [*srh-11p::tax-4 cDNA::sl2e::TagRFP-t; vha-6p::gfp*]
ZXW970	*tax-4(ks28)* III; *hkdEx970* [*gcy-8p::tax-4 cDNA::sl2e::TagRFP-t; vha-6p::gfp*]
ZXW971	*tax-4(ks28)* III; *hkdEx971* [*gcy-8p::tax-4 cDNA::sl2e::TagRFP-t; vha-6p::gfp*]
ZXW972	*tax-4(ks28)* III; *hkdEx972* [*gcy-8p::tax-4 cDNA::sl2e::TagRFP-t; vha-6p::gfp*]
ZXW973	*hkdEx973* [*srh-11p::R-GECO1.0; lin-44p::gfp*]
ZXW974	*daf-11(m47)* V; *hkdEx974* [*srh-11p::R-GECO1.0; lin-44p::gfp*]
ZXW975	*daf-11(m47)* V; *hkdEx975* [*daf-11p::daf-11::sl2e::TagRFP-t; srh-11p::G-GECO1.1; lin-44p::gfp*]
ZXW976	*hkdEx976* [*sra-9p::R-GECO1.0; lin-44p::gfp*]
ZXW977	*daf-11(m47)* V; *hkdEx977* [*sra-9p::R-GECO1.0; lin-44p::gfp*]
ZXW978	*daf-11(m47)* V; *hkdEx978* [*daf-11p::daf-11::sl2e::TagRFP-t; sra-9p::G-CaMP2.0; lin-44p::gfp*]
ZXW979	*hkdEx979* [*sra-9p::R-GECO1.0; lin-44p::gfp*]
